# A Molecular Electron Density Theory Study of the Domino Reaction of *N*-Phenyl Iminoboranes with Benzaldehyde Yielding Fused Bicyclic Compounds

**DOI:** 10.3390/molecules28176211

**Published:** 2023-08-23

**Authors:** Luis R. Domingo, María José Aurell, Mar Ríos-Gutiérrez

**Affiliations:** Department of Organic Chemistry, University of Valencia, Dr. Moliner 50, 46100 Burjassot, Spain; domingo@utopia.uv.es (L.R.D.); maria.j.aurell@uv.es (M.J.A.)

**Keywords:** MEDT, iminoborane, benzaldehyde, electrophilic aromatic substitution

## Abstract

The reaction of *N*-phenyl iminoborane with benzaldehyde yielding a fused aromatic compound, recently reported by Liu et al., has been studied within the Molecular Electron Density Theory (MEDT). Formation of the fused aromatic compound is a domino process that comprises three consecutive reactions: (i) formation of a weak molecular complex between the reagents; (ii) an intramolecular electrophilic attack of the activated carbonyl carbon of benzaldehyde on the *ortho* position of the *N*-phenyl substituent of iminoborane; and (iii) a formal 1,3-hydrogen shift yielding the final fused aromatic compound. The two last steps correspond to a Friedel–Crafts acylation reaction, the product of the second reaction being the tetrahedral intermediate of an electrophilic aromatic substitution reaction. However, the presence of the imino group adjacent to the aromatic ring strongly stabilizes the corresponding intermediate, being the reaction product when the *ortho* positions are occupied by t-butyl substituents. This domino reaction shows a great similitude with the Brønsted acid catalyzed Povarov reaction. Although *N*-phenyl iminoborane can experience a formal [2+2] cycloaddition reaction with benzaldehyde, its higher activation Gibbs free energy compared to the intramolecular electrophilic attack of the activated carbonyl carbon of benzaldehyde on the *ortho* position of the *N*-phenyl substituent, 6.6 kcal·mol^−1^, prevents the formation of the formal [2+2] cycloadduct. The present MEDT study provides a different vision of the molecular mechanism of these reactions based on the electron density.

## 1. Introduction

**Scheme 1:**
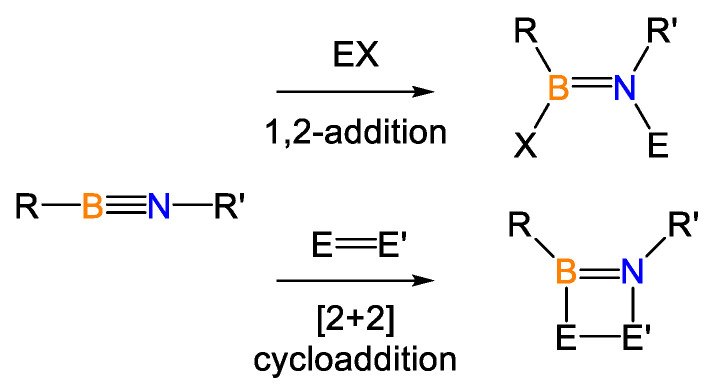
Reactions of iminoboranes.

Iminoboranes are isoelectronic with alkynes yet contain a polar B–N triple bond, which readily undergoes 1,2-addition [1,2] or [2+2] cycloaddition reactions [3,4,5] (see Scheme 1).

**Scheme 2:**
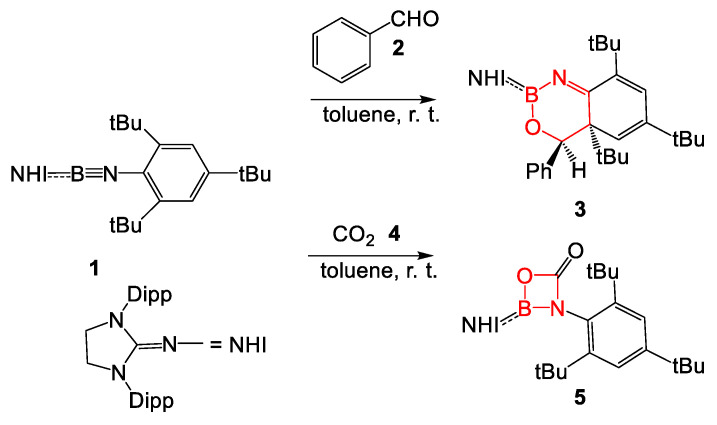
Experimental reactions of *B*-(*N*-heterocyclic imine) *N*-aryl iminoborane **1** with benzaldehyde **2** and CO_2_ **4**, reported by Liu et al. [6].

Recently, Liu et al. [6] suggested that BN-substituted π-conjugated systems may be versatile species participating in formal Diels-Alder reactions, given that enynes have been well documented as the diene component for [4+2] cycloaddition reactions with alkenes [7,8,9]. Thus, when *B*-(*N*-heterocyclic imine) *N*-aryl iminoborane **1** was treated with benzaldehyde **2** in toluene at room temperature, the fused bicyclic compound **3** was obtained with a yield of 97% (see Scheme 2) [6]. On the other hand, when the *N*-aryl iminoborane **1** was treated with CO_2_, the four-membered heterocyclic **5** was obtained with a yield of 65% (see Scheme 2).

Liu et al. theoretically studied the two reactions, suggesting that the formation of fused bicyclic compound **3** takes place by a concerted [4+2] cycloaddition reaction between *N*-aryl iminoborane **1** and benzaldehyde **2** through a transition state structure (TS) that presented an activation Gibbs free energy of 23.1 kcal·mol^−1^ [6]. The formal [2+2] cycloaddition of *N*-aryl iminoborane **1** with benzaldehyde **2** to give an adduct like **5** was also studied. The TS associated with the corresponding formal [2+2] cycloaddition reaction was found to be 9.6 kcal·mol^−1^ above the TS associated with the formation of fused bicyclic compound **3**, explaining the absence of the formal [2+2] cycloadduct.

**Scheme 3:**
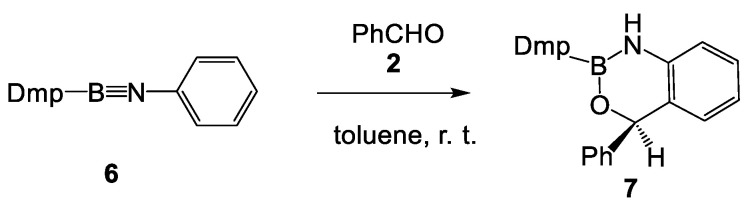
Experimental reaction of *N*-phenyl iminoborane **6** with benzaldehyde **2**, reported by Liu et al. [6].

Interestingly, when *N*-phenyl iminoborane **6**, which has no substitution at the *ortho* positions of benzene, was treated with benzaldehyde **2**, the aromatic compound **7** was obtained with a yield of 97% (see Scheme 3) [6]. Formation of the aromatic compound **7** was explained by a hydrogen shift from the *ortho* position of the formal [4+2] cycloadduct to the imino nitrogen atom.

Formation of the aromatic compound **7** can be understood as an intramolecular boron-catalyzed Friedel–Crafts acylation reaction of the *N*-phenyl iminoborane **6**. The presence of alkyl substituents at the *ortho* positions of the *N*-aryl iminoborane **1** prevents the elimination of the alkyl group, while the presence of strong electron-releasing amino nitrogen at the aromatic compound strongly stabilizes the tetrahedral intermediate involved in an electrophilic aromatic substitution (S_E_Ar) reaction, thus yielding the non-aromatic compound **3**.

**Scheme 4:**
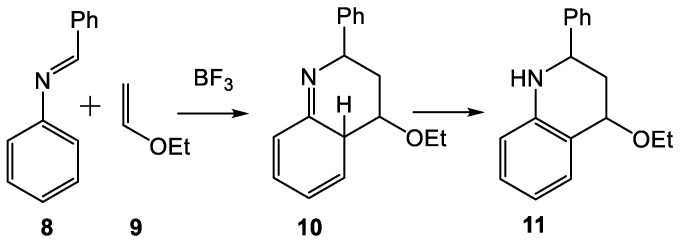
Povarov reaction of *N*-aryl imines with nucleophilic ethylenes.

The reaction between an *N*-aryl imine **8** and a nucleophilic ethylene **9** in the presence of a Lewis acid, known as the Povarov reaction [10], has become a powerful tool for the construction of the 1,2,3,4-tetrahydroquinoline (THQ) structure (see Scheme 4). Povarov reactions are domino processes involving two consecutive reactions: (i) a Lewis acid (LA) catalyzed aza-Diels-Alder (A-DA) reaction between an *N*-aryl imine **8** and a nucleophilic ethylene **9** giving a formal [4+2] cycloadduct **10**; and (ii) a subsequent 1,3-hydrogen shift in **10** yielding the final THQ **11**, with regeneration of the aromatic ring [11].

**Scheme 5:**
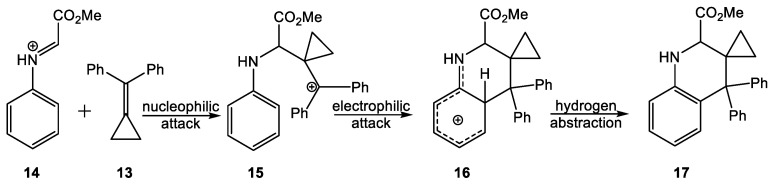
Brønsted acid catalyzed Povarov reaction.

In 2015, the mechanism of the Brønsted acid-catalyzed Povarov reaction of *N*-phenyl-*C*-methoxycarbonyl imine **12** with methylenecyclopropane **13** was investigated [12]. The quick nucleophilic attack of methylenecyclopropane **13** on the carbon atom of protonated imine **14** yields the stabilized carbocation intermediate **15**, which experiences a rapid electrophilic attack on the *ortho* position of the aromatic ring of the *N*-phenyl imine (see Scheme 5). Finally, a hydrogen abstraction on the tetrahedral cationic intermediate **16** yields the aromatic THQ **17**, a formal [4+2] cycloadduct. The mechanism of this Brønsted acid-catalyzed Povarov reaction was explained as a domino process initialized by the formation of a cationic intermediate, which experiences a quick intramolecular Friedel–Crafts alkylation reaction yielding the final THQ **17** [12].

A comparative study of the reaction products of iminoboranes **1** and **6** given in Scheme 2 and Scheme 3 with the species theoretically characterized in the study of the Brønsted acid catalyzed Povarov reaction given in Scheme 5 shows a great similitude. The formal [4+2] cycloadduct **17** is obtained using an intramolecular Friedel–Crafts alkylation reaction in the strong electrophilic cation intermediate **15**.

**Scheme 6:**
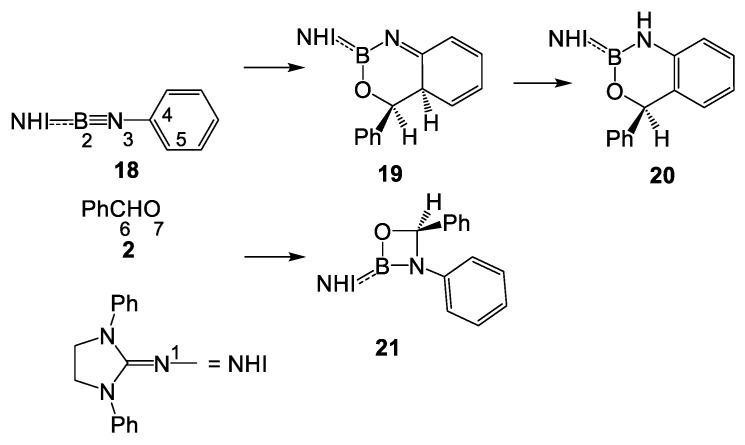
Reactions of model *N*-phenyl iminoborane **18** with benzaldehyde **2**, are investigated in the present theoretical study.

In order to provide a new mechanistic interpretation of the reaction of *N*-aryl iminoborane **1** with benzaldehyde **2**, experimentally and theoretically studied by Liu et al. (see Scheme 2) [6], a Molecular Electron Density Theory (MEDT) [13] study of the reaction of model *N*-phenyl iminoborane **18** with benzaldehyde **2**, yielding fused aromatic compound **20** and the formal [2+2] cycloadduct **21**, is herein studied. In model *N*-phenyl iminoborane **18**, the Dipp groups of experimental *N*-phenyl iminoborane **6** were substituted by phenyl groups (see Scheme 6).

## 2. Results and Discussion

### 2.1. ELF and AIM Characterization of the Electronic Structure of N-Phenyl Iminoborane ***18*** and Benzaldehyde ***2***

**Figure 1:**
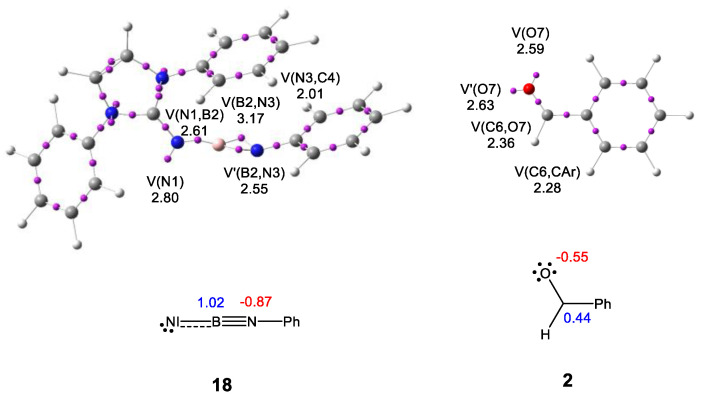
*ω*B97X-D/6-311G(d,p) ELF basin attractor positions, populations of the most relevant valence basins, natural atomic charges, and ELF Lewis-like structures of *N*-phenyl iminoborane **18** and benzaldehyde **2**. Valence basin populations and natural atomic charges are given in an average number of electrons, e. Negative charges are colored in red, and positive charges in blue.

The topological analysis of the electron localization function (ELF) [14] permits a quantitative characterization of the electron density distribution in a molecule [15], allowing the establishment of a correlation between its electronic structure and its reactivity. Thus, an ELF topological analysis of the electronic structures of *N*-phenyl iminoborane **18** and benzaldehyde **2** was performed first. ELF basin attractor positions, together with the most relevant valence basin populations, as well as ELF Lewis-like structures, are shown in Figure 1. Atom numbering is provided in Scheme 6.

ELF topology of the iminoborane moiety of **18** shows the presence of one V(N1) monosynaptic basin, integrating 2.80 e, one V(N1,B2) disynaptic basin, integrating 2.61 e, a pair of disynaptic basins, V(B2,N3) and V′(B2,N3), integrating a total of 5.72 e, and one V(N3,C4) disynaptic basin, integrating 2.01 e. While the two V(B2,N3) and V′(B2,N3) disynaptic basins allow characterizing the B2–N3 bonding region as a B2–N3 triple bond, the V(N1,B2) disynaptic basin allows relating the N1–B2 bonding region to a populated N1–B2 single bond. The V(N1) monosynaptic basin indicates the presence of a non-bonding region at the N1 nitrogen. Finally, the population of the V(N3,C4) disynaptic basin, which is near 2.0 e, permits the relation of the N3–C4 bonding region as an N3–C4 single bond. The presence of the V(N1,B2) and V(B2,N3) disynaptic basins suggests that the B2 boron is covalently bound to the N1 and N3 nitrogens.

The carbonyl C–O bonding region of benzaldehyde **2** is characterized by the presence of one V(C6,O7) disynaptic basin, integrating 2.36 e, and a pair of monosynaptic basins, V(O7) and V′(O7), integrating a total of 5.22 e. The low population of the V(C6,O7) disynaptic basin, which is a consequence of the strong electronegative character of the O7 oxygen, characterizes the C6–O7 bonding region as a C6–O7 single bond.

Finally, a natural population analysis (NPA) [16,17] of the atomic charges of *N*-phenyl iminoborane **18** indicates that while the B2 boron is positively charged by +1.02 e, the N3 nitrogen is negatively charged by −0.87 e. The NPA of the atomic charges of benzaldehyde **2** indicates that while the carbonyl C6 carbon is positively charged by +0.44 e, the O7 oxygen is negatively charged by −0.55 e.

Interestingly, while the ELF analysis of the electronic structure of *N*-phenyl iminoborane **18** agrees with the proposed Lewis structures for iminoboranes given in Scheme 1, the NPA gives opposite charges at the boron and nitrogen to those proposed for them on the basis of classic Lewis structures.

**Figure 2:**
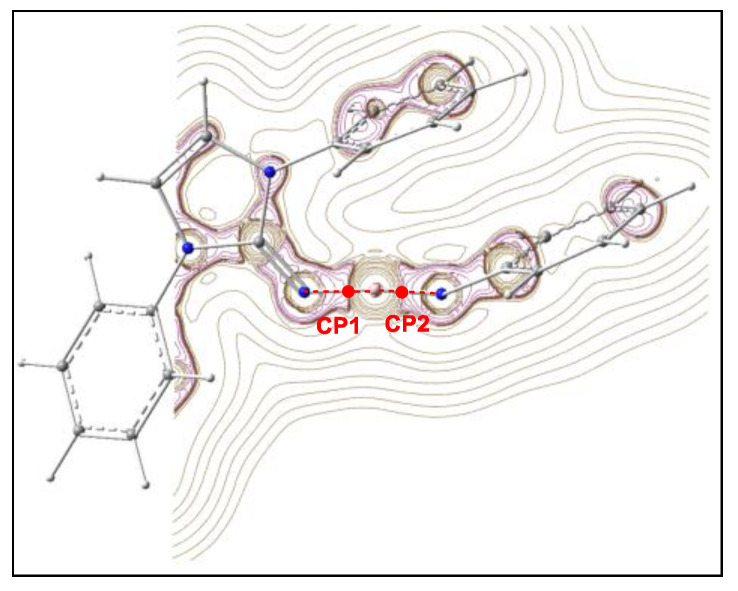
Representations of the contour line maps of the Laplacian ∇^2^*ρ*(r) of the electron density at *N*-phenyl iminoborane **18**. The critical points **CP1** and **CP2** associated with the N1–B2 and B2–N3 interaction regions, respectively, are colored in red.

In order to further understand the nature of the electronic interactions at the N1–B2 and B2–N3 bonding regions of *N*-phenyl iminoborane **18**, a topological analysis of the electron density in these N–B bonding regions was performed within the Quantum Theory of Atoms in Molecules (QTAIM) [18,19]. The contour line map of the Laplacian ∇^2^*ρ*(r) of the electron density of *N*-phenyl iminoborane **18** is shown in Figure 2, while the QTAIM parameters at the (3, −1) critical points (CPc) **CP1** and **CP2** associated with the N–B regions are given in Table 1.

QTAIM analysis of *N*-phenyl iminoborane **18** provides electron density *ρ*(r) values higher than 0.22 e·Å^−3^ for both **CP1** and **CP2**, suggesting covalent interactions [20]. However, both CPs present a high positive Laplacian ∇^2^*ρ*(r) value and a negative local energy density H(r), suggesting an intermediate interaction such as highly polar covalent bonds or dative bonds (see Table 1) [20,21,22,23,24]. The H(r)/*ρ*(r) << 0 and G(r)/*ρ*(r) > 1 found at **CP1** and **CP2** are also characteristic of intermediate interactions.

Espinosa [21] proposed a useful criterion to characterize interactions at the CPs using an analysis of the ratio of the potential and kinetic energy densities, |V(r)|/G(r). For non-covalent interactions, |V(r)|/G(r) < 1, partially covalent interactions are characterized by 1 < |V(r)|/G(r) < 2, while covalent interactions show |V(r)|/G(r) > 2. The calculated values of |V(r)|/G(r) at **CP1** and **CP2** of *N*-phenyl iminoborane **18** are given in Table 1. The |V(r)|/G(r) values of **CP1** and **CP2** are lower than 1.68, indicating that these B–N bonding regions present somewhat covalent character, which may well correspond to a dative bond type considering the previous parameters. This suits the common understanding that boron usually participates as an acceptor in chemical bonding with donors such as nitrogen.

Recently, Berski et al. [25] studied the nature of the B–N multiple bonds by using ELF and QTAIM, finding a similar behavior for the B–N triple and single bonds to that found in *N*-phenyl iminoborane **18**. While ELF showed the presence of V(B,N) disynaptic basins integrating ca. 6.4 and 2.0 e, the QTAIM analysis yielded the typical features for shared (∇^2^*ρ*(r) < 0) and closed-shell (∇^2^*ρ*(r) > 0) interactions. The ELF and natural bond orbital (NBO) topological analyses yielded a similar description for the B–N bonds. Both approaches revealed a significant contribution of the nitrogen atom to the B–N bonding, which is in agreement with a formal dative mechanism of its formation. Consequently, unlike the C–C or N–N covalent bonds, the N→B bonds should be considered as dative bonds [25].

### 2.2. Study of the Reaction of N-Phenyl Iminoborane ***18*** with Benzaldehyde ***2***

**Scheme 7:**
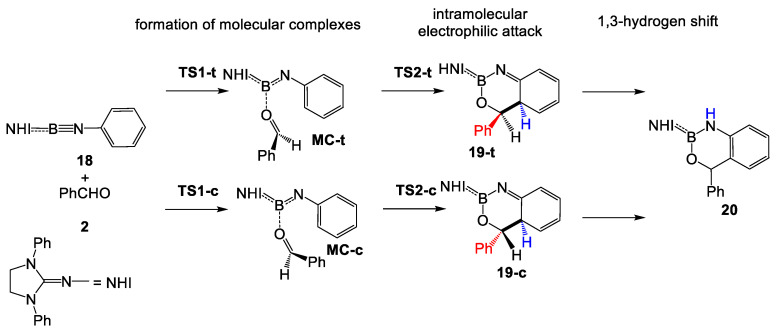
*Trans* and *cis* stereoisomeric reaction paths associated with the domino reaction of *N*-phenyl iminoborane **18** with benzaldehyde **2**.

The reaction of *N*-phenyl iminoborane **18** with benzaldehyde **2** yielding aromatic compound **20** is a domino process that comprises three consecutive reactions: (i) formation of a weak molecular complex (MC) resulting from the interactions between the carbonyl O7 oxygen of benzaldehyde **2** and the B2 boron of *N*-phenyl iminoborane **18**; (ii) an intramolecular electrophilic attack of the activated carbonyl C6 carbon of benzaldehyde **2** on the *ortho* C5 carbon of the phenyl substituent of iminoborane **18** yielding the fused bicyclic compound **19**; and finally (iii) a formal 1,3-hydrogen shift from the C5 carbon to the N3 nitrogen regenerating the aromatic ring (see Scheme 7). In the cases of *N*-aryl iminoboranes having alkyl substituents in the *ortho* positions, this process is not possible, obtaining compound **19** as the reaction product (see Scheme 2). This domino process can take place along two stereoisomeric reaction paths, named *trans* and *cis*, depending on the relative position of the phenyl substituent of benzaldehyde **2** with respect to the *ortho* hydrogen of the *N*-phenyl substituent of iminoborane **18** (see Scheme 7). The relative enthalpies and Gibbs free energies are given in Table 2, while complete thermodynamic data are gathered in Table S2 in Supporting Information. 

The activation Gibbs free energies associated with the formation of the MCs are 15.8 (**TS1-c**) and 16.7 (**TS1-t**) kcal·mol^−1^, their formation being endergonic by 14.9 (**MC-c**) and 17.2 (**MC-t**) kcal·mol^−1^. The very high endergonic character of these MCs means that they cannot be considered reaction intermediates. Note, however, that the formation of these MCs is necessary for the electrophilic activation of the carbonyl C6 carbon of benzaldehyde **2** for the subsequent electrophilic attack to the phenyl substituent of *N*-phenyl iminoborane **18**.

From these MCs, the subsequent electrophilic attacks of the activated carbonyl C6 carbon of benzaldehyde **2** on the *ortho* C5 carbon of the phenyl substituent of *N*-phenyl iminoborane **18** have low activation Gibbs free energies of 6.2 (**TS2-c**) and 2.6 (**TS2-t**) kcal·mol^−1^. From the separated reagents, the formation of the fused bicyclic compounds **19-c** and **19-t** is exergonic by 6.8 and 4.6 kcal·mol^−1^, respectively. Finally, a subsequent formal 1,3-hydrogen shift converts the fused bicyclic compounds **19** into the aromatic compound **20**. This process is strongly exergonic by 39.4 kcal·mol^−1^ as a consequence of the regeneration of the aromatic ring of the phenyl substituent. Due to the strain associated with this intramolecular 1,3-hydrogen shift process, it has a very high activation energy [26], and consequently, this tautomerization requires an external acid/base species [27].

**Figure 3:**
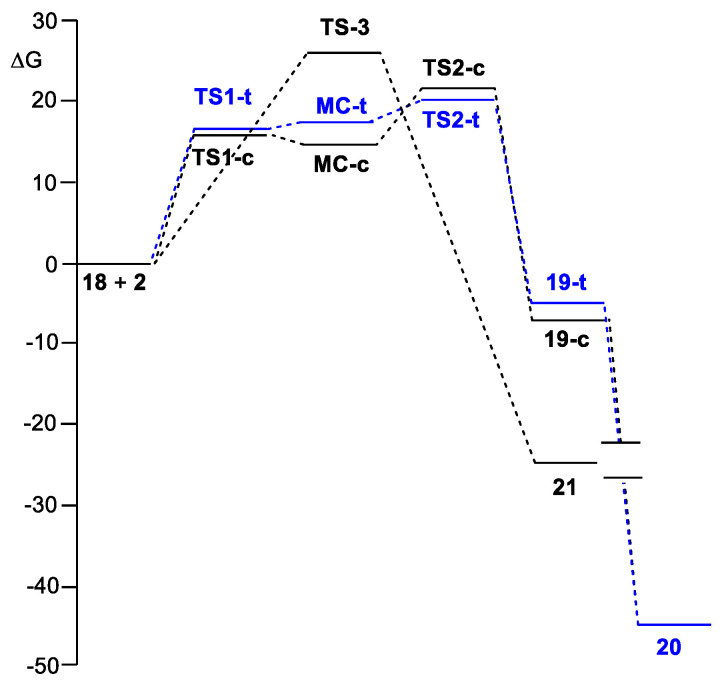
*ω*B97X–D/6-311G(d,p) Gibbs free energy profiles, in kcal·mol^−1^, computed in toluene at 298.15 K, of the competitive reaction paths associated with the domino reaction of *N*-phenyl iminoborane **18** with benzaldehyde **2**. The most favorable *trans* reaction path yielding the aromatic compound **20** is given in blue.

Considering the endergonic character of the formation of MCs, the second step, corresponding to the electrophilic attack of the carbonyl C6 carbon of benzaldehyde **2** on the *ortho* C5 carbon of the phenyl substituent of *N*-phenyl iminoborane **18**, can be considered the rate-determining step (RDS) of this domino processes. If the formation of these fused bicyclic compounds is considered irreversible, *trans* **19-t** will be the product of a kinetic control as **TS2-t** is 1.8 kcal·mol^−1^ below **TS2-c** (see Figure 3). Consequently, the activation Gibbs free energy associated with this domino reaction via **TS2-t** is 19.8 kcal·mol^−1^.

**Scheme 8:**
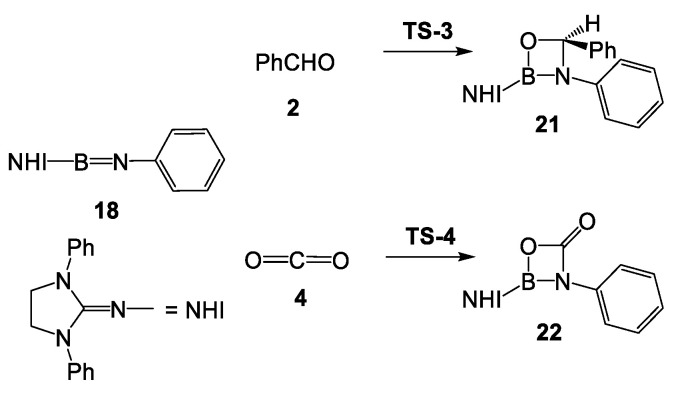
Formal [2+2] cycloaddition reaction between *N*-phenyl iminoborane **18** with benzaldehyde **2** and with CO_2_ **4**.

Liu et al. found that when *N*-aryl iminoborane **1** was treated with CO_2_, the four-membered heterocyclic compound **5** was obtained with a yield of 65% (see Scheme 2) [6]. However, the formation of a formal [2+2] cycloadduct **21** between *N*-aryl iminoborane **1** and formaldehyde **2** was not experimentally observed. Therefore, the two formal [2+2] cycloaddition reactions in *N*-phenyl iminoborane **18** were studied herein (see Scheme 8). Relative enthalpies and Gibbs free energies are given in Table 2, while complete thermodynamic data are gathered in Table S2 in Supporting Information. 

The activation Gibbs free energy associated with the [2+2] addition of CO_2_ **4** to *N*-phenyl iminoborane **18** via **TS-4** is 19.4 kcal·mol^−1^ (see Table 2), formation of the formal [2+2] cycloadduct **22** being strongly exergonic by 14.1 kcal·mol^−1^. The activation Gibbs free energy associated with this [2+2] addition is close to that associated with the intramolecular electrophilic attack in **MC-t**, justifying the experimental observation of cycloadduct **22** (see Table 2). On the other hand, the activation Gibbs free energy associated with the [2+2] addition of benzaldehyde **2** to *N*-phenyl iminoborane **18** via **TS-3** is 26.4 kcal·mol^−1^, formation of the formal [2+2] cycloadduct **21** being also strongly exergonic by 24.4 kcal·mol^−1^. Consequently, although the formation of the formal [2+2] cycloadduct **21** is thermodynamically more favorable than the formation of fused bicyclic compounds **19**, the former reaction is 6.6 kcal·mol^−1^ kinetically more unfavorable, thus preventing the formation of the formal [2+2] cycloadduct **21**.

**Figure 4:**
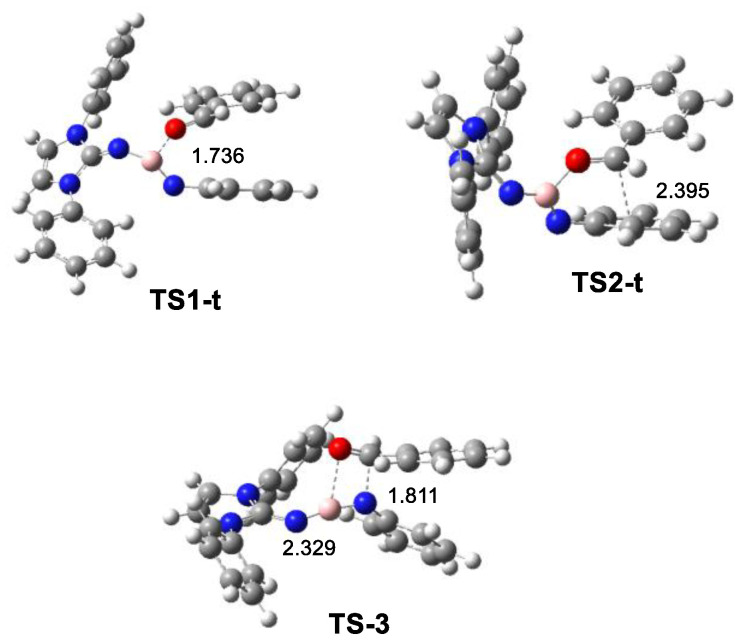
*ω*B97X-D/6-311G(d,p) optimized geometries in toluene of the TSs involved in the *trans* reaction paths and formal [2+2] cycloaddition between *N*-phenyl iminoborane **18** with benzaldehyde **2**.

The geometries of the TSs involved in the more favorable *trans* reaction path are given in Figure 4. At the TS related to the formation of **MC-t**, the B2–O7 distance is 1.736 Å, while this distance at **MC-t** is 1.629 Å, indicating the very advanced character of **TS1-t**. At **TS2-t**, the C5–C6 distance is 2.395 Å. Considering that this distance at **MC-t** is 2.843 Å, **TS2-t** can be considered very early, in agreement with the low activation energy associated with **TS2-t**, 2.6 kcal·mol^−1^. Finally, at **TS-3**, associated with the formation of the formal [2+2] cycloadduct **21**, the N3–C6 and B2–O7 distances are 1.811 and 2.329 Å, respectively. These distances indicate that it corresponds to a highly asynchronous TS associated with the nucleophilic attack of the N3 nitrogen of *N*-phenyl iminoborane **18** on the carbonyl C6 carbon atom of benzaldehyde **2**.

Finally, the polar character of the [2+2] cycloaddition reactions was evaluated by computing the global electron density transfer (GEDT) [28] at the corresponding TSs. TSs with a GEDT lower than 0.05 e correspond to non-polar processes, while TSs with a GEDT higher than 0.20 e correspond to high-polar processes. The GEDT computed at the two TSs is 0.25 e at **TS-3** and 0.27 e at **TS-4**. These high values account for the high polar character of these formal [2+2] cycloaddition reactions, which can be related to the nucleophilic attack of the imino N3 nitrogen of *N*-phenyl iminoborane **18** on the carbonyl carbons of benzaldehyde **2** and CO_2_. In addition, the higher polar character of **TS-4** than that of **TS-3** accounts for the lower activation enthalpy of the former (see Table 2).

### 2.3. ELF and QTAIM Analysis of the Stationary Points Associated with the Trans Reaction Path of the Domino Reaction between N-Phenyl Iminoborane ***18*** and Benzaldehyde ***2***

**Figure 5:**
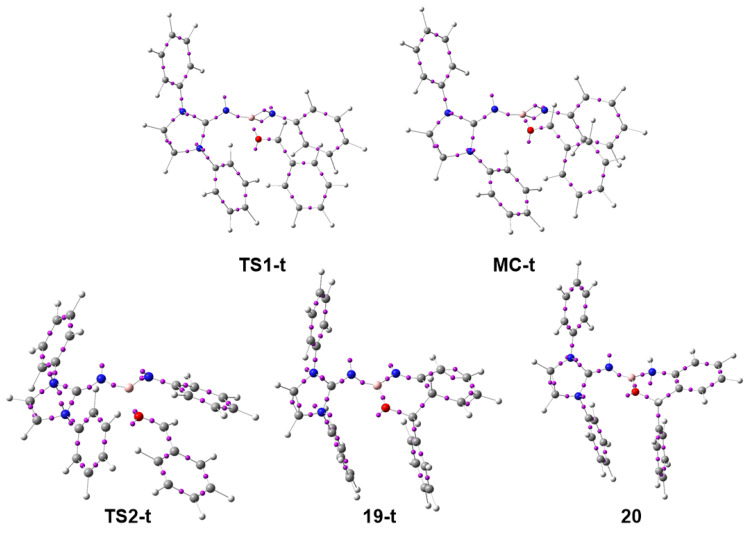
*ω*B97X-D/6-311G(d,p) ELF basin attractor positions of the stationary points associated with the *trans* reaction path of the domino reaction between *N*-phenyl iminoborane **18** and benzaldehyde **2**.

In order to reveal the structural changes along the domino reaction between *N*-phenyl iminoborane **18** and benzaldehyde **2** yielding the aromatic compound **20**, an ELF topological analysis of the stationary points in toluene associated with the more favorable *trans* reaction path was performed. ELF basin attractor positions are shown in Figure 5, while the most relevant ELF valence basin populations are given in Table 3.

Some appealing conclusions can be obtained from the variation of ELF valence basin populations corresponding to the stationary points involved in the *trans* reaction path: (i) Along the domino reaction, some molecular regions are depopulated while others are populated. Thus, while the N1–B2 is depopulated from 2.74 e at **TS1-t** to 2.48 e at **20** the N3=C4 bonding region is populated from 2.00 e at **TS1-t** to 2.75 e at **19-t**. At the final aromatic compound **20**, the population of the N3–C4 bonding region is 1.91 e. The total population of the non-bonding region of the O7 oxygen decreases from 5.22 e at benzaldehyde **2** to 4.30 e at **20**; (ii) the presence of benzaldehyde **2** at **MC-t**, with the O7 oxygen near the B2 boron, remarkably depopulates the B2–N3 region from 5.72 to 3.15 e, and causes the accumulation of non-bonding electron density at the N3 nitrogen; (iii) the absence of any V(B2,O7) disynaptic basin at **MC-t** indicates that at this weak MC, there is a non-covalent interaction between the B2 boron and the carbonyl O7 oxygen; and finally, (iv) the bonding changes found in the conversion of **19-t** into the aromatic compound **20** via the 1,3-hydrogen shift are a consequence of the strong stabilization of the tetrahedral compound **19-t** resulting from the intramolecular electrophilic attack of benzaldehyde **2** on the *ortho* position of *N*-phenyl iminoborane **18**.

**Figure 6:**
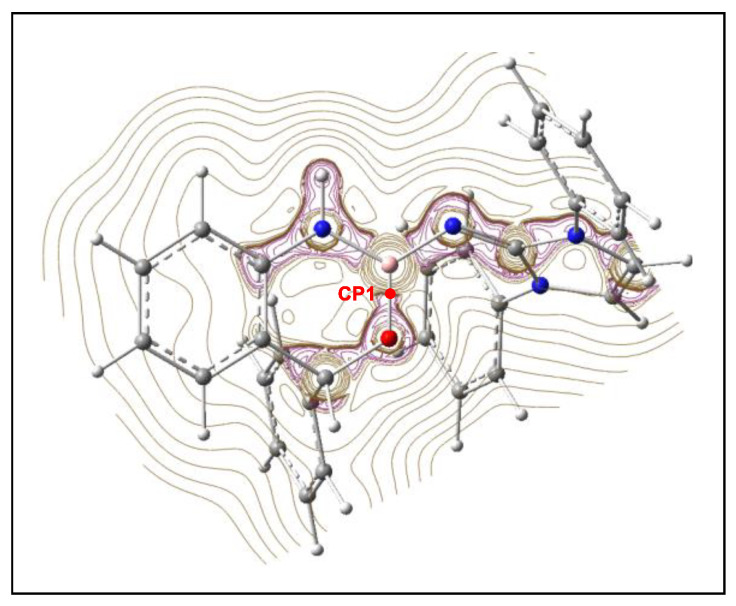
Representations of the contour line maps of the Laplacian ∇^2^*ρ*(r) of the electron density at the aromatic compound **20**. The critical point **CP1** associated with the B2–O7 interaction region is colored in red.

Finally, a QTAIM topological analysis of the electron density *ρ*(r) at the stationary points **MC-t**, **19-t**, and **20** was performed in order to characterize the nature of the B2–O7 and C5–C6 interactions at these species. The calculated QTAIM parameters at the (3, −1) CPs at these interacting regions, **CP1** and **CP2**, are given in Table 4, while the contour line map of the Laplacian ∇^2^*ρ*(r) of the electron density of the final aromatic compound **20** is shown in Figure 6.

QTAIM analysis of these species shows very different values of electron density at **CP1**. While **MC-t** presents a *ρ*(r) value of 0.09 e·Å^−3^, this increases up to 0.19 e·Å^−3^ at products **19-t** and **20**. These values suggest an intermediate (dative) B2–O7 interaction at **MC-t** that increases its covalent character at both **19-t** and **20**. On the other hand, the value of ca. 0.25 e·Å^−3^ at **CP2** is characteristic of a C5–C6 covalent bond.

At the three compounds, **CP1** shows a positive Laplacian ∇^2^*ρ*(r) value, indicating the absence of any pure covalent interaction in the B2–O7 bonding region (see Table 4). Conversely, at **19-t** and **20**, **CP2** shows a negative Laplacian ∇^2^*ρ*(r) value, indicating the presence of a covalent C5–C6 interaction.

The G(r)/*ρ*(r) and |V(r)|/G(r) parameters also allow a clear distinction between the two different types of bonds in these species. At the three compounds, G(r)/*ρ*(r) > 1 at **CP1**, clearly indicating an intermediate (dative) B2–O7 interaction, while G(r)/*ρ*(r) < 1 at **CP2**, featuring the covalent C5–C6 bond at **19-t** and **20**. On the other hand, the |V(r)|/G(r) values of **CP1** at the three compounds are lower than 1.42, indicating that, according the Espinosa criteria [21], the B2–O7 bonding region of these species present a non-covalent interaction with somewhat covalent character, while the corresponding values of **CP2** at **19-t** and **20**, higher than 4.40, indicate a covalent interaction between the C5 and C6 carbons.

The H(r)/*ρ*(r) indicators are always negative, thus providing no particular discrimination. However, the similar QTAIM parameters at each **CP1** and **CP2** in both **19-t** and **20** indicate no remarkable changes in the B2–O7 and C5–C6 bonding regions along the conversion of **19-t** into the aromatic compound **20**.

On the other hand, the figure of the contour line map of the Laplacian ∇^2^*ρ*(r) of the electron density of the aromatic compound **20** in the plain containing the N1 and N3 nitrogen and the O7 oxygen nuclei shows that these heteroatoms are not covalently bound to the B2 boron (see Figure 6).

Recently, Berski et al. [29] studied the nature of chalcogen B-Ch (Ch = O, S, Se, Te) multiple bonds, finding that the B–O bond presents similar behaviors to the B–N ones. The B–O bond has a high degree of polarity, with a large positive ∇^2^*ρ*(r) value. The oxygen quantum atom delivers about 90% of the electron density of the V(B,O) disynaptic basin, indicating that the O→B bond has a high dative character. Thus, it is reasonable that the B–N bonds are also dative bonds, as Figure 6 suggests.

### 2.4. BET Study of the Molecular Mechanisms of the Electrophilic Attack of Benzaldehyde ***2*** on the Phenyl Substituent of Imine Borane ***18***

**Scheme 9:**
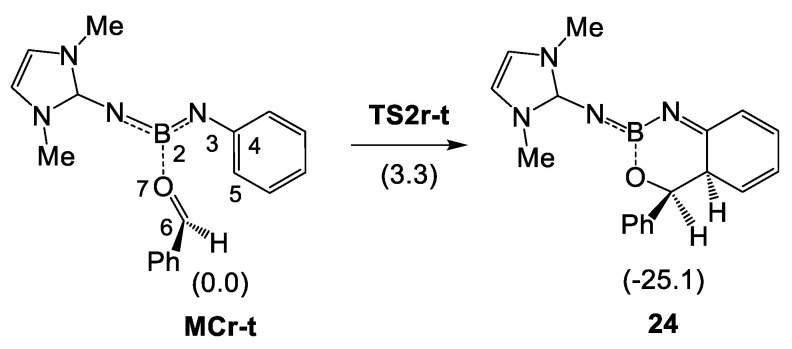
Step of the electrophilic attack of the carbonyl C6 carbon on the *ortho* C5 carbon of the phenyl substituent of *N*-phenyl iminoborane **23**. Relative energies in toluene are given in parentheses in kcal·mol^−1^.

**Figure 7:**
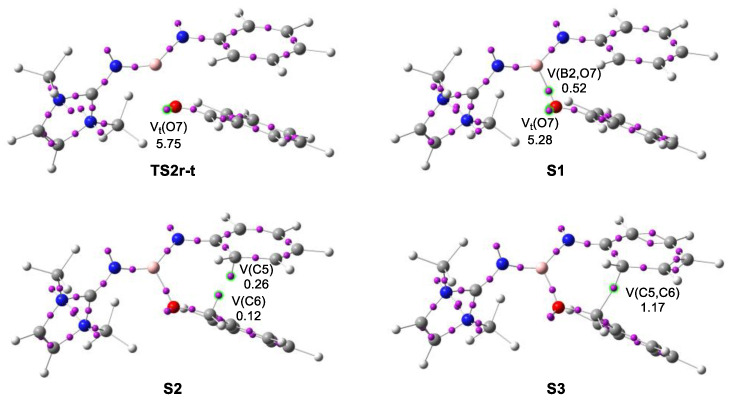
ELF basin attractor positions of **TS2r-t** and the structures involved in the formation of the C5–C6 single bond.

**Scheme 10:**
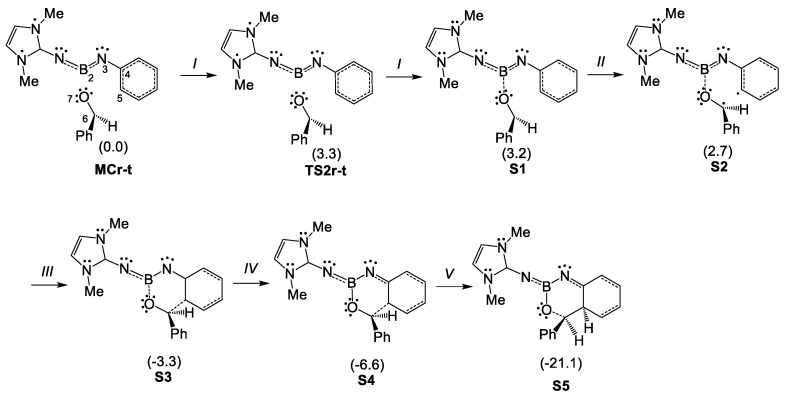
Representation of the molecular mechanism of the electrophilic attack of the carbonyl C6 carbon on the *ortho* C5 carbon of the phenyl substituent of *N*-phenyl iminoborane **23**, by Lewis-like structures based on the topological analysis of the ELF.

In order to characterize the bonding changes taking place in the first step of the acylation of imine borane **18** with benzaldehyde **2**, a Bonding Evolution Theory (BET) [30] study of the electron density redistribution along the electrophilic attack of the carbonyl C6 carbon of benzaldehyde **2** on the *ortho* C5 carbon of the phenyl substituent of *N*-phenyl iminoborane **23**, as a reduced model of *N*-phenyl iminoborane **18**, was carried out (see Scheme 9). In *N*-phenyl iminoborane **23**, the two phenyl substituents present in *N*-phenyl iminoborane **18** were replaced by methyl groups. The topological analysis of the ELF of *N*-phenyl iminoborane **23** is given in Figure S1 in Supporting Information. The population of the most relevant regions at key structures of the IRC is given in Table 5, while a simplified representation of the mechanism is displayed in Scheme 10. The ELF valence basin attractor positions of the structures involved in the bond formation processes are shown in Figure 7.

The first structure of the IRC corresponds to the molecular complex **MCr-t** formed between the two interacting frameworks. Similar to **MC-t** (see Table 3), the B2–N3 bonding region is characterized as an underpopulated double bond integrating 3.03 e, with a non-bonding electron density of 2.66 e at the N3 nitrogen. No conjugation of the B2–N3 bond with the phenyl ring is observed, given that the N3–C4 region presents a population of 2.02 e. On the other hand, the benzaldehyde moiety presents a markedly polarized carbonyl C6–O7 single bond integrating 2.16 e, while the O7 oxygen atom bears a total population of 5.41 e.

The topological analysis of the ELF of **TS2r-t** shows no significant changes in the electron density distribution with respect to that of **MCr-t**, which accounts for the low activation energy associated with the electrophilic attack of the carbonyl C6 carbon of the benzaldehyde framework on the *ortho* C5 carbon of the phenyl substituent of *N*-phenyl iminoborane one, 3.3 kcal·mol^−1^. This barrier might be associated with the depopulation of the B2–N3 bonding region by 0.29 e towards the N3 nitrogen and N3–C4 regions and with the depopulation of the C6–O7 bonding region by 0.27 e towards the O7 oxygen. Note that the V(O7) monosynaptic basins have been populated by 0.34 e.

Just after passing **TS2r-t**, the topological analysis of the ELF suggests that the non-covalent interaction between the carbonyl O7 oxygen and the B2 boron becomes covalent at a B2–O7 distance of ca. 1.59 Å and an initial bonding population of 0.52 e, through the donation of non-bonding electron density of the carbonyl O7 oxygen to the B2 boron (see the new V(B2,O7) disynaptic basin at **S1** in Figure 7 and the corresponding Lewis-like structure in Scheme 10). However, QTAIM analysis suggests an intermediate dative-type bond.

At structure **S2**, the progressive depopulation of the C4–C5 and C6–O7 bonding regions gathers non-bonding electron density at the C5 and C6 carbon atoms, with a population of 0.26 and 0.12 e, respectively, which are associated with two C5 and C6 *pseudoradical* centers [31] (see the new V(C5) and V(C6) monosynaptic basins in **S2** in Figure 7 and the corresponding Lewis-like structure in Scheme 10).

At **S3**, the C-to-C coupling of these two C5 and C6 *pseudoradical* centers forms the new C5–C6 single bond at a C–C distance of ca. 2.00 Å, and an initial population of 1.17 e, via the sharing bond formation model [28] (see the V(C5,C6) disynaptic basin at **S3** in Figure 7 and the corresponding Lewis-like structure in Scheme 10). At this moment, the C4–C5 bonding region can already be considered a single bond after the continued population along the reaction path, just as the C4–C and C5–C regions of the new hexadiene ring (see the Lewis-like structure of **S3** in Scheme 10). Only at the end of the reaction path does the N3–C4 region become an imine partial N–C double bond, in agreement with the expected structure for cycloadduct **24** (see **S4** in Scheme 10).

Some appealing conclusions can be drawn from this BET study: (i) formation of the new C5–C6 single bond takes place at a C–C distance of ca. 2.00 Å, following the general sharing model involving C–C multiple bonds [28]; (ii) a delocalized structure in the phenyl ring, such as that in a cationic tetrahedral intermediate characteristic of the first step of S_E_Ar reactions, is not observed. Instead, a localized and well-defined cyclohexadienimine structure is formed as a consequence of the presence of the N2 nitrogen in *N*-phenyl iminoborane **23**. Note that the fused bicyclic compound **24** is 25.1 kcal·mol^−1^ more stable than **23** (see Scheme 9). This behavior allows explaining the stability of fused bicyclic compound **3** in which the *ortho* positions of the aromatic ring are substituted by a t-butyl group (see Scheme 2).

### 2.5. REG-IQA Energy Partitioning Analysis of the Activation Energy

As mentioned above, no significant changes in the bonding pattern are observed at **TS2r-t** with respect to **MCr-t** (see both Lewis-like structures in Scheme 10). Thus, the activation energy associated with the electrophilic attack of carbonyl C6 carbon of benzaldehyde **2** on the *ortho* C5 carbon of the phenyl substituent of *N*-phenyl iminoborane **23** is only 3.3 kcal·mol^−1^. Based on variations of ELF valence basin populations, it seems that this low activation barrier is mainly associated with the depopulation of the B2–N3 and C6–O7 bonding regions. However, changes in ELF populations should not be directly related to the energy of the system [32]. Thus, in order to determine the origin of the activation energy, a REG-IQA energy partitioning analysis was carried out (see the theoretical background in Supporting Information for more details on REG-IQA). The ten IQA energy terms with the largest contribution to the barrier, i.e., REG absolute values, are gathered in Table 6, while Pearson correlation coefficients of REG regressions are given in Table S1 in Supporting Information. 

The REG-IQA analysis shows that the terms participating the most in the activation energy are of an interatomic nature. The terms most contributing towards the barrier, i.e., the most unfavorable energies for the reaction, are V*_inter_*(C6,O7) and V*_inter_*(B2,N3), associated with the interatomic interactions that take place in the C6–O7 and B2–N3 bonding regions as they are depopulated. The REG values of the corresponding $V_{cl}^{AB}$ and $V_{xc}^{AB}$ terms indicate that these interatomic interactions are mainly related to classical electrostatic (non-covalent) interactions rather than covalency. Note that, especially in the B2–N3 region, the REG value of V*_cl_*(B2,N3) is almost four times larger than that of V*_xc_*(B2,N3).

On the other hand, the factors most working against the barrier, i.e., the ones that most favor the reaction, are V*_inter_*(B2,O7) and V*_inter_*(N3,C4), associated with the interatomic interactions in the B2–O7 and N3–C4 bonding regions, followed by V*_inter_*(C5,C6), belonging to one of the phenyl ring bonds. While the electrostatic component of V*_inter_*(B2,O7) and V*_inter_*(N3,C4) overcomes the effect of exchange-correlation in decreasing the activation barrier, the favorable role of V*_inter_*(C5,C6) in the barrier is mostly due to exchange-correlation, i.e., covalency.

Consequently, the present REG-IQA study allows concluding that the low activation energy associated with the electrophilic attack of carbonyl C6 carbon on the *ortho* C5 carbon of the phenyl substituent of *N*-phenyl iminoborane **23** is mainly associated with the unfavorable electrostatics developed in the B2–N3 and C6–O7 bonding regions as a consequence of their depopulation from **MCr-t** to **TS2r-t**.

## 3. Computational Methods

All calculations were performed using the *ω*B97X-D [33] functional together with the standard 6-311G(d,p) basis set [34]. The TSs were characterized by the presence of only one imaginary frequency. The Berny method was used in optimizations [35,36]. The intrinsic reaction coordinates (IRC) paths [37] were traced to establish the unique connection between the TSs and the corresponding minima [38,39].

Solvent effects of toluene in the thermodynamic calculations were taken into account by full optimization of the gas-phase structures at the same computational level using the polarizable continuum model (PCM) [40,41] in the framework of the self-consistent reaction field (SCRF) [42,43,44]. Values of *ω*B97X-D/6-311G(d,p) enthalpies, entropies, and Gibbs free energies in toluene were calculated with standard statistical thermodynamics [34] at room temperature and pressure by PCM frequency calculations at the solvent-optimized structures.

The GEDT [28] values were computed using the equation GEDT(f) = Σq_f_, where q is the natural charges [16,17] of the atoms belonging to one of the two frameworks (f) at the TS geometries.

The Gaussian 16 suite of programs was used to perform the calculations [45]. ELF [14] analyses of the *ω*B97X-D/6-311G(d,p) monodeterminantal wavefunctions were performed by using the TopMod [46] package with a cubical grid of step size of 0.1 Bohr. Molecular geometries and ELF basin attractors were visualized by using the GaussView program [47].

A stand-alone script based on the Ramer-Douglas-Peucker algorithm [48,49] was used to find a reduced but still suitable number of structures on which to run the REG-IQA [50] analysis out of the 23 points belonging to the respective activation IRC path; an RMSE value of 0.01 kcal·mol^−1^ was considered. The IQA analysis [51] was performed with the AIMAll package [52] using the corresponding B3LYP/6-311G(d,p) monodeterminantal wavefunctions. To achieve this, the corresponding TSs were optimized, and the IRCs were obtained at that computational level. Note that the *ω*B97X-D functional is not available in AIMAll. The REG analysis was carried out via the REG.py program developed by Popelier’s group [53].

## 4. Conclusions

The reaction of *N*-phenyl iminoborane **18** with benzaldehyde **2** yielding fused aromatic compound **20**, recently reported experimentally and theoretically by Liu et al. [6], has been studied within MEDT at the *ω*B97X-D/6-311G(d,p) computational level.

**Scheme 11:**
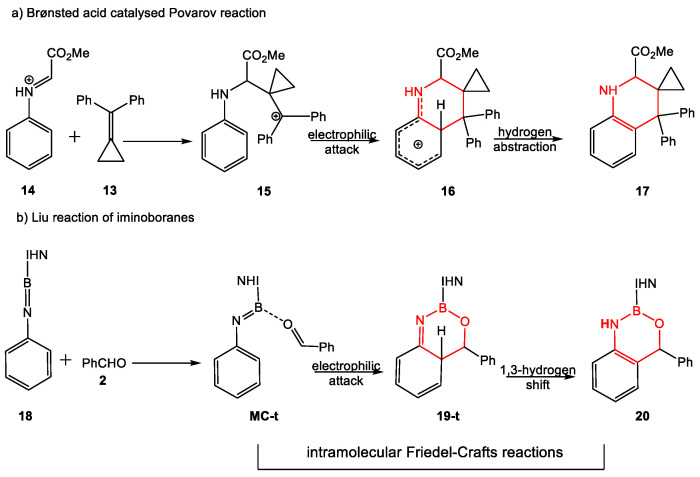
Comparative analysis of the domino reactions involved in the Brønsted acid-catalyzed Povarov reaction and the reaction of N-phenyl iminoborane **18** with benzaldehyde **2**.

Formation of the fused aromatic compound **20** is a domino process that comprises three consecutive reactions: (i) formation of a weak molecular complex **MC-t** between the *N*-phenyl iminoborane **18** and benzaldehyde **2**; (ii) an intramolecular electrophilic attack of the activated carbonyl carbon of benzaldehyde on the *ortho* position of the *N*-phenyl substituent yielding the fused bicyclic compound **19-t**; (iii) and a formal 1,3-hydrogen shift yielding the final fused aromatic compound **20**. The two last reactions of this domino process correspond to an intramolecular Friedel–Crafts acylation reaction, the fused bicyclic compound **19-t** of the second reaction being the tetrahedral intermediate of a S_E_Ar reaction. The presence of the imino N3 nitrogen adjacent to the aromatic ring strongly stabilizes the tetrahedral compound **19-t**, being obtained as the reaction product when the *ortho* positions of the phenyl substituent are occupied by t-butyl groups. This domino reaction shows a great similitude with the mechanism of the Brønsted acid-catalyzed Povarov reactions (see Scheme 11).

Although *N*-phenyl iminoborane **18** can experience a formal [2+2] cycloaddition reaction with benzaldehyde **2**, the higher activation Gibbs free energy associated with this cycloaddition reaction, 26.4 kcal·mol^−1^, than that associated with the intramolecular electrophilic attack of the activated carbonyl carbon of benzaldehyde on the *ortho* position of the *N*-phenyl substituent, 19.8 kcal·mol^−1^, prevents the formation of the formal [2+2] cycloadduct **21**.

Analysis of the electron density reorganization along the domino reaction of *N*-phenyl iminoborane **23** with benzaldehyde **2** allows for an explanation of the experimental outcomes. The electron density changes taking place along the electrophilic attack of the carbonyl C6 carbon of benzaldehyde **2** on the *ortho* position of the phenyl substituent permits a strong stabilization of the product of the electrophilic attach, allowing this compound as the reaction product when the subsequent formal 1,3-hydrogen shift is not possible. ELF and QTAIM analyses of the electron density of the species involved in this domino reaction indicate that the N→B and O→B bonds have a dative nature instead of a covalent one.

The present MEDT study provides a different vision of the molecular mechanism of these reactions to that reported by Liu et al., allowing an interpretation of this domino reaction based on the changes in electron density. The MCs are very unfavorable coordination complexes between the carbonyl oxygen atom of benzaldehyde and the boron of the *N*-phenyl iminoboranes, while the second reaction of this domino process is the first step of an intramolecular S_E_Ar reaction related to a Friedel–Crafts acylation. In contrast, Liu et al. related **TS1-t** to the TS of a concerted DA reaction [6].

## Figures and Tables

**Table 1:** QTAIM parameters at the (3, −1) CPs associated with the N1–B2 and B2–N3 interacting regions of *N*-phenyl iminoborane **18**. *ρ*(r) is given in e·Å^−3^, ∇^2^*ρ*(r) in e·Å^−5^, and H(r), G(r), and V(r) in hartree·Å^−3^.

	CP1 (N1–B2)	CP2 (B2–N3)
Density *ρ*(r)	0.2284	0.2648
Laplacian ∇^2^*ρ*(r)	0.4435	0.9547
G(r)	0.3463	0.5161
V(r)	−0.5817	−0.7936
H(r)	−0.2354	−0.2775
G(r)/*ρ*(r)	1.5160	1.9484
H(r)/*ρ*(r)	−1.0306	−1.0473
|V(r)|/G(r)	1.6797	1.5376

**Table 2:** *ω*B97X-D/6-311G(d,p) relative enthalpies and Gibbs free energies (in kcal·mol^−1^), computed in toluene at 298.15 K, of the stationary points involved in the domino reaction of *N*-phenyl iminoborane **18** with benzaldehyde **2**.

	ΔH	ΔG
**TS1-c**	0.3	15.8
**TS1-t**	1.7	16.7
**MC-c**	0.7	14.9
**MC-t**	2.3	17.2
**TS2-c**	4.0	21.1
**TS2-t**	2.7	19.8
**19-c**	−22.6	−6.8
**19-t**	−21.3	−4.6
**20**	−62.8	−45.1
**TS-3**	11.1	26.4
**21**	−40.1	−24.4
**TS-4**	7.8	19.4
**22**	−27.5	−14.1

**Table 3:** ELF basin populations, in average number of electrons, e, of the stationary points involved in the *trans* reaction path of the domino reaction between *N*-phenyl iminoborane **18** and benzaldehyde **2**.

	18	2	TS1-t	MC-t	TS2-t	19-t	20
V(N1,B2)	2.61		2.74	2.44	2.43	2.53	2.48
V(N1)	2.80		2.74	2.74	2.75	2.78	2.85
V(B2,N3)	3.17		1.45	1.33	2.78	2.27	2.72
	2.55		1.80	1.82			
V(N3,H)							2.14
V(N3)			2.48	2.55	2.72	2.62	1.19
V(N3,C4)	2.01		2.00	2.01	2.18	2.75	1.91
V(C4,C5)	2.75		2.67	2.65	2.53	2.07	2.79
V(C5,C6)						1.9	2.08
V(C6,O7)		2.36	2.10	2.05	1.82	1.36	1.33
V(O7)		2.63	2.81	2.89	3.17	4.28	4.3
V(O7)		2.59	2.66	2.66	2.65		
V(B2,O7)						2.01	2.02

**Table 4:** AIM parameters, in atomic units a.u., of the (3, −1) CPs at the stationary points **MC-t**, **19-t**, and **20** in the regions associated with the B2–O7 and C5–C6 interacting atoms. *ρ*(r) is given in e·Å^−3^, ∇^2^*ρ*(r) in e·Å^−5^, and H(r), G(r), and V(r) in hartree·Å^−3^.

	MC-t	19-t		20	
	CP1 (B2–O7)	CP1 (B2–O7)	CP2 (C5–C6)	CP1 (B2–O7)	CP2 (C5–C6)
Density *ρ*(r)	0.0938	0.1833	0.2374	0.1902	0.2533
Laplacian ∇^2^*ρ*(r)	0.3036	0.8081	−0.5244	0.8242	−0.6088
G(r)	0.1316	0.3405	0.0547	0.3538	0.0571
V(r)	−0.1872	−0.4791	−0.2406	−0.5015	−0.2664
H(r)	−0.0557	−0.1386	−0.1858	−0.1476	−0.2093
G(r)/*ρ*(r)	1.4030	1.8576	0.2304	1.8601	0.2254
H(r)/*ρ*(r)	−0.5938	−0.7561	−0.7826	−0.7760	−0.8263
|V(r)|/G(r)	1.4228	1.4070	4.3983	1.4176	4.6626

**Table 5:** ELF valence basin populations, relative energies in toluene, and B2–O7 and C5–C6 distances of the most relevant structures along the IRC associated with the electrophilic attack of the carbonyl C6 carbon on the *ortho* C5 carbon of the phenyl substituent of *N*-phenyl iminoborane **23**. Populations are given in average number of electrons, e, energies in kcal·mol-1, and distances in angstroms, Å.

Structures	MCr-t	TS2r-t	S1	S2	S3	S4	S5
Phases		*I*	*II*	*III*	*IV*	*V*	
d(B2–O7)	1.657	1.630	1.586	1.542	1.471	1.456	1.418
d(C5–C6)	2.829	2.461	2.39	2.284	1.995	1.906	1.575
ΔE	0.0	3.3	3.2	2.7	−3.3	−6.6	−21.1
V(N1,B2)	2.66	2.43	2.58	2.56	2.56	2.55	2.55
V(B2,N3)	3.03	2.74	2.68	2.61	2.45	2.41	2.28
V(N3)	2.66	2.77	2.77	2.75	2.66	2.63	2.61
V(N3,C4)	2.02	2.19	2.23	2.30	2.53	2.59	2.74
V(C4,C5)	2.65	2.76	2.78	2.55	2.29	2.23	2.08
V(C6,O7)	2.16	1.89	1.83	1.73	1.52	1.49	1.37
V(O7)	5.41 *	5.75 *	5.28 *	5.10 *	4.74 *	4.61	4.40
V(B2,O7)			0.52	0.81	1.39	1.53	1.88
V(C5)				0.26			
V(C6)				0.12			
V(C5,C6)					1.17	1.34	1.81

* the asterisk superscript indicates the presence of two V(X) and V′(X) monosynaptic basins; only the sum of their population is included.

**Table 6:** Ten largest REG*_i_* values corresponding to the IQA partitioning into intra-atomic $E_{intra}^{A}$ and interatomic $V_{inter}^{AB}$ energy terms along the activation IRC path associated with the S_E_Ar reaction between *N*-phenyl iminoborane **23** and benzaldehyde **2**. REG*_i_* values of the $V_{cl}^{AB}$ and $V_{xc}^{AB}$ contributors to $V_{inter}^{AB}$ are also included. Relative energies are given in kcal·mol^−1^.

Energy Term	REG	$\mathrm{REG}\;V_{cl}^{AB}$	$\mathrm{REG}\;V_{xc}^{AB}$
V*_inter_*(C6,O7)	72.42	50.74	21.68
V*_inter_*(B2,N3)	60.39	47.69	12.71
V*_inter_*(B2,C4)	30.40	30.36	0.04
V*_inter_*(N3,C6)	21.61	21.67	−0.06
V*_inter_*(N1,O7)	18.28	20.43	−2.16
V*_inter_*(C4,O7)	−22.44	−21.79	−0.65
*E_intra_*(C6)	−31.07		
V*_inter_*(C5,C6)	−38.14	−2.30	−35.83
V*_inter_*(N3,C4)	−56.85	−35.99	−20.85
V*_inter_*(B2,O7)	−103.97	−92.58	−11.39

## Data Availability

The data underlying this study are available in the published article and its supporting information.

## Supplementary Materials

The following supporting information can be downloaded at: https://www.mdpi.com/article/10.3390/molecules28176211/s1, Theoretical background on REG-IQA; Figure S1: *ω*B97X-D/6-311G(d,p) ELF basin attractor positions, populations of the most relevant valence basins, and natural atomic charges of *N*-phenyl iminoborane **23**; Table S1: Pearson correlation coefficients *R* of the ten IQA terms with largest REG*_i_* values along the activation IRC path associated with the S_E_Ar reaction between *N*-phenyl iminoborane **23** and benzaldehyde **2**; Table S2: *ω*B97X-D/6-311G(d,p) total electronic energies, enthalpies, entropies, and Gibbs free energies, computed in toluene at 298.15 K, of the stationary points involved in the reactions of *N*-phenyl iminoborane **18** with benzaldehyde **2** and with CO_2_ **4**. *ω*B97X-D/6-311G(d,p) Cartesian coordinates of the optimized geometries in toluene of the stationary points involved in the reactions of *N*-phenyl iminoborane **18** with benzaldehyde **2**, and with CO_2_ **4**, including the single imaginary frequencies for the TSs. References [54,55,56,57] are cited in the supplementary materials.
