# Supplementary material for: A Molecular Electron Density Theory Study of the Domino Reaction of N-Phenyl Iminoboranes with Benzaldehyde Yielding Fused Bicyclic Compounds
Source: Molecules. 2023 Aug 23;28(17):6211. doi: 10.3390/molecules28176211 (PMC10488853; doi:10.3390/molecules28176211)
Supplement: Supplementary file 1 [file molecules-28-06211-s001.zip › molecules-2577006-supplementary.pdf]

Supplementary Material

A Molecular Electron Density Theory Study of the Domino Reaction of *N*-Phenyl Iminoboranes with Benzaldehyde Yielding Fused Bicyclic Compounds

Luis R. Domingo, María José Aurell and Mar Ríos-Gutiérrez *

Department of Organic Chemistry, University of Valencia, Dr. Moliner 50, 46100 Burjassot, Valencia, Spain;
domingo@utopia.uv.es (L.R.D.); maria.j.aurell@uv.es (M.J.A.)
* Correspondence: m.mar.rios@uv.es

Index

S2 Theoretical background on REG-IQA

S4 **Figure S1** with the ωB97X-D/6-311G(d,p) ELF basin attractor positions, populations of the most relevant valence basins, and natural atomic charges of *N*-phenyl iminoborane **23**.

S5 **Table S1** with the Pearson correlation coefficients *R* of the twelve IQA terms with largest REG_i values along the activation IRC path associated with the S_EAr reaction between *N*-phenyl iminoborane **23** and benzaldehyde **2**.

S6 **Table S2** with the ωB97X-D/6-311G(d,p) total electronic energies, enthalpies, entropies, and Gibbs free energies, computed in toluene at 298.15 K, of the stationary points involved in the reactions of *N*-phenyl iminoborane **18** with benzaldehyde **2**, and with CO_2 **4**.

S7 ωB97X-D/6-311G(d,p) Cartesian coordinates of the optimized geometries in toluene of the stationary points involved in the reactions of *N*-phenyl iminoborane **18** with benzaldehyde **2**, and with CO_2 **4**, including the single imaginary frequencies for the TSs.

Theoretical background on REG-IQA

The IQA scheme divides the total energy into two main energy contributions: the intra-atomic energy, E_{intra}^{A}, and the interatomic energy, V_{inter}^{AB} [1]. The interatomic energy is in turn divided into two additional terms: the interatomic electrostatic energy (typically referred to as "classical"), V_{cl}^{AB}, and the interatomic exchange-correlation energy, V_{xc}^{AB}, in such a way that $V_{inter}^{AB} = V_{cl}^{AB} + V_{xc}^{AB}$. While E_{intra} has been associated with steric effects [2], V_{cl} is related to electrostatic interactions [3,4], and V_{xc} quantifies covalency [5]. The REG method [6] compares the gradient of a given energy contribution E_i against the gradient of the total energy E_{total}, using linear regression: $E_i(s) = m_{REG,i}E_{total}(s) + c_i$, where s is the control coordinate (IRC in our case) governing the change in the system, $m_{REG,i}$ is the REG value itself, and c_i is the y-intercept, which has currently no known chemical meaning. Thus, the REG method is only valid when the Pearson correlation coefficient R is close to 1 (these values for the studied reactions are given in Table S1). The REG values allow a semi-quantitative interpretation of the individual energy terms contributions to the total energy. Both the sign and the magnitude of the REG value are important. In summary, (energy) terms E_i with large positive REG_i values contribute the most to the total behaviour of the system, while terms with large negative REG_i values work against the total behaviour of the system. For instance, in the activation energy path, energy terms with positive REG values are unfavourable and contribute to the barrier, while those with negative REG values are favourable and facilitate the reaction. Ranking the energies from largest to smallest produces an ordered list of IQA energy terms that directly contribute towards (or against) a given energy segment, which in our case coincides with a barrier. This list provides a chemically intuitive interpretation for each energy segment being considered.

References

1. Blanco, M. A.; Martín Pendás, A.; Francisco, E. Interacting Quantum Atoms: A Correlated Energy Decomposition Scheme Based on the Quantum Theory of Atoms in Molecules, *J. Chem. Theory Comput.* **2005**, *1*, 1096–1109.
2. Wilson, A. L.; Popelier, P. L. A. Exponential Relationships Capturing Atomistic Short-Range Repulsion from the Interacting Quantum Atoms (IQA) Method, J. Phys. Chem. A **2016**, *120*, 9647–9659.
3. Popelier, P. L. A.; Kosov, D. S. Atom–atom partitioning of intramolecular and intermolecular Coulomb energy, *J. Chem. Phys.* **2001**, *114*, 6539–6547.
4. Popelier, P. L. A.; Joubert, L.; Kosov, D. S. Convergence of the Electrostatic Interaction Based on Topological Atoms, *J. Phys. Chem. A*, **2001**, *105*, 8254–8261.
5. Martín Pendás, A.; Francisco, E.M.; Blanco, A.; Gatti, C. Bond Paths as Privileged Exchange Channels, *Chem. Eur. J.* **2007**, *13*, 9362–9371.
6. Thacker, J. C. R.; Popelier, P. L. A. The ANANKE relative energy gradient (REG) method to automate IQA analysis over configurational change, *Theor. Chem. Acc.* 2017, **136**, 86.

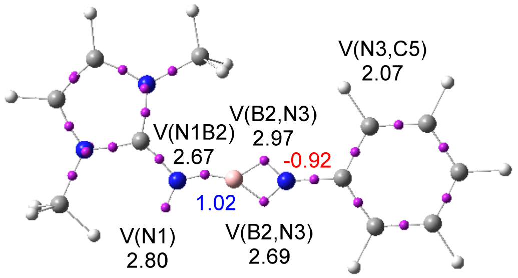

Figure S1. ωB97X-D/6-311G(d,p) ELF basin attractor positions, populations of the most relevant valence basins, and natural atomic charges of N-phenyl iminoborane **23**. Valence basin populations and natural atomic charges are given in average number of electrons, e. Negative charges are coloured in red, and positive charges in blue.

Table S1. Pearson correlation coefficients *R* of the twelve IQA terms with largest REG_i values along the activation IRC path associated with the S_EAr reaction between *N*-phenyl iminoborane **23** and benzaldehyde **2**.

Region	V_{inter}^{AB}	V_{cl}^{AB}	V_{xc}^{AB}
C6,O7	0.96	0.95	0.97
B2,N3	0.97	0.98	0.97
B2,C4	0.97	0.97	0.93
N3,C6	0.97	0.97	-0.34
N1,O7	0.98	0.98	-0.98
C4,O7	-0.97	-0.97	-0.98
C6*	-0.96	-	-
C5,C6	-0.96	-1.00	-0.95
N3,C4	-0.96	-0.96	-0.97
B2,O7	-0.97	-0.97	-0.97

* R value for E_{intra}(C6)

Table S2. ωB97X-D/6-311G(d,p) Total electronic energies, E in a.u., enthalpies, H in a.u., entropies, S in cal mol^{-1} K^{-1}, and Gibbs free energies, ◎G in a.u., computed in toluene at 298.15 K, of the stationary points involved in the reactions of *N*-phenyl iminoborane **18** with benzaldehyde **2**, and with CO_2 **4**.

	E	H	S	G
**2**	-345.538539	-345.420657	79.4	-345.458381
**18**	-1054.262393	-1053.899597	160.0	-1053.975616
**TS1-c**	-1399.801877	-1399.319748	187.6	-1399.408863
**TS1-t**	-1399.798699	-1399.317466	189.3	-1399.407430
**MC-c**	-1399.801895	-1399.319208	191.6	-1399.410250
**MC-t**	-1399.798820	-1399.316514	189.7	-1399.406635
**TS2-c**	-1399.795757	-1399.313907	182.1	-1399.400440
**TS2-t**	-1399.797591	-1399.315952	182.2	-1399.402503
**19-c**	-1399.840957	-1399.356312	186.2	-1399.444765
**19-t**	-1399.839278	-1399.354264	183.3	-1399.441359
**20**	-1399.906705	-1399.420291	180.0	-1399.505814
**TS-3**	-1399.784561	-1399.302506	188.3	-1399.391984
**21**	-1399.869452	-1399.384130	186.9	-1399.472937
**4**	-188.577984	-188.562531	51.0	-188.586773
**TS-4**	-1242.828290	-1242.449645	172.4	-1242.531549
**22**	-1242.887385	-1242.506023	165.8	-1242.584809

ωB97X-D/6-311G(d,p) Cartesian coordinates of the optimized geometries in toluene of the stationary points involved in the reactions of *N*-phenyl iminoborane **18** with benzaldehyde **2**, and with CO_2 **4**, including the single imaginary frequencies for the TSs.

2

6	-1.941520	0.460696	0.001758
8	-2.740196	-0.110659	-0.698547
1	-2.272223	1.240807	0.718514
6	-0.483302	0.206171	-0.004124
6	0.062592	-0.752217	-0.860095
6	0.344052	0.930263	0.852074
6	1.428575	-0.981603	-0.855803
1	-0.599427	-1.303599	-1.517759
6	1.713732	0.699804	0.855766
1	-0.087569	1.674063	1.514993
6	2.253149	-0.255575	0.002020
1	1.856889	-1.724603	-1.518329
1	2.358905	1.261727	1.520686
1	3.322005	-0.437153	0.003403

18

7	3.660279	-0.414703	0.094884
7	2.146547	0.856841	1.020335
7	-1.163733	-0.386862	-0.896642
7	1.437152	-0.688964	-0.688858
6	4.305239	-1.416337	-0.680439
6	-2.391981	0.145175	-0.629339
6	5.245855	-2.240671	-0.069979
6	4.295067	0.373037	1.056283
6	2.315469	-0.123840	0.070957
6	3.364759	1.155102	1.623195
6	-4.880037	1.309197	-0.012401
6	-3.191644	-0.366181	0.403818
6	-2.885359	1.229345	-1.370598
6	-4.413523	0.214608	0.709549
6	-4.108869	1.804723	-1.059148
6	0.883993	1.449803	1.327710
6	5.912858	-3.198450	-0.822336

6	0.415455	2.501328	0.549450
6	-0.839643	3.034222	0.810817
6	0.119891	0.934536	2.367527
6	4.018603	-1.555811	-2.034958
6	-1.132170	1.477251	2.624384
6	-1.613228	2.521300	1.844361
6	4.680019	-2.527149	-2.773414
6	5.630354	-3.346345	-2.174496
5	0.091271	-0.485800	-0.745210
1	5.357351	0.319323	1.215203
1	3.444761	1.911966	2.383484
1	-5.008186	-0.191827	1.521297
1	-4.463540	2.649753	-1.640420
1	6.645329	-3.838224	-0.344314
1	-1.222500	3.837594	0.193521
1	-1.742376	1.068423	3.420627
1	-2.605823	2.916787	2.022901
1	4.452848	-2.638756	-3.827119
1	6.145278	-4.099802	-2.758734
1	-2.822020	-1.211518	0.973127
1	-5.835853	1.760349	0.228470
1	-2.278818	1.620630	-2.179619
1	3.276089	-0.918832	-2.495049
1	5.443829	-2.142468	0.991131
1	1.024533	2.876242	-0.264668
1	0.500402	0.103253	2.949412

TS1-c

Imaginary frequency -114.1676 cm-1

8	0.000000	0.000000	0.000000
7	0.000000	0.000000	4.210797
7	1.687960	0.000000	2.808207
7	-0.318289	1.227328	2.212207
7	-0.233862	2.576186	0.029058
6	0.395635	0.495593	2.973167
6	1.013058	-0.785047	4.758851
6	2.042801	-0.788091	3.904574
6	2.553166	0.274298	1.720006
6	2.779281	1.585293	1.309359
6	3.627594	1.828729	0.235737
6	4.264258	0.777123	-0.413584

6	4.044521	-0.529559	0.009823
6	3.184103	-0.784245	1.069487
6	-1.288308	0.153635	4.782048
6	-1.874795	-0.929419	5.433591
6	-3.118331	-0.785351	6.034517
6	-3.789161	0.429440	5.971960
6	-3.204848	1.501934	5.307589
6	-1.955363	1.374926	4.716820
6	-0.067914	2.659545	-1.321971
6	-0.920079	2.007493	-2.240692
6	-0.686422	2.061244	-3.609498
6	0.396209	2.766626	-4.120593
6	1.232797	3.443591	-3.233023
6	1.004969	3.399003	-1.867572
6	0.760694	-0.070536	-0.968733
5	-0.190851	1.537559	0.853745
1	0.918993	-1.228150	5.734397
1	3.020889	-1.228864	3.986284
1	3.792552	2.848647	-0.089319
1	4.927084	0.975225	-1.247619
1	4.530397	-1.356097	-0.495244
1	-3.567573	-1.632047	6.540618
1	-4.763556	0.538787	6.433501
1	-3.722405	2.452511	5.250224
1	-1.359189	1.539389	-4.282762
1	2.073257	4.015880	-3.613822
6	0.720335	-1.160415	-1.928634
6	1.598418	-1.121041	-3.013825
6	-0.182058	-2.218437	-1.783700
6	1.575299	-2.139401	-3.954891
1	2.282979	-0.285839	-3.117732
6	-0.198452	-3.233749	-2.723571
1	-0.857372	-2.224810	-0.936580
6	0.679162	-3.192801	-3.806485
1	2.248668	-2.112794	-4.802883
1	-0.893874	-4.058066	-2.621026
1	0.660698	-3.989320	-4.541691
1	1.521364	0.702060	-1.120274
1	1.655920	3.927335	-1.179517
1	0.577154	2.805068	-5.188591
1	-1.766303	1.450910	-1.851853
1	-1.367556	-1.886957	5.456531
1	-1.502276	2.203929	4.192490

1	2.285297	2.403896	1.816264
1	2.982643	-1.801656	1.384588

TS1-t

Imaginary frequency -162.2460 cm-1

8	0.000000	0.000000	0.000000
7	0.000000	0.000000	4.903039
7	2.062982	0.000000	4.190785
7	0.392379	-0.070686	2.519221
7	-0.838602	1.949289	1.473721
6	0.754191	-0.001518	3.747324
6	0.836090	0.002911	6.018572
6	2.103830	0.015157	5.580746
6	3.185830	0.096900	3.325947
6	4.088451	1.141447	3.490323
6	5.185060	1.240353	2.641968
6	5.365380	0.312714	1.623454
6	4.453112	-0.724730	1.461802
6	3.364910	-0.841794	2.314741
6	-1.418228	-0.104463	4.966693
6	-1.981248	-1.063961	5.803860
6	-3.362359	-1.163763	5.902506
6	-4.177147	-0.322900	5.153080
6	-3.604379	0.622841	4.311022
6	-2.223648	0.747074	4.219555
6	-1.107603	2.728459	0.389312
6	-1.968152	2.313033	-0.648861
6	-2.180893	3.106354	-1.772891
6	-1.551426	4.336043	-1.902835
6	-0.718617	4.776949	-0.871894
6	-0.505351	3.998011	0.249288
6	0.336005	0.559913	-1.040845
6	1.371161	1.582966	-1.153607
6	2.266084	1.826316	-0.106213
6	3.201215	2.838210	-0.229801
6	3.244103	3.610415	-1.389371
6	2.364308	3.362315	-2.436905
6	1.433153	2.341292	-2.323769
5	-0.205150	0.794736	1.637584
1	-0.160280	0.258483	-1.974253
1	0.438816	0.012571	7.018251

1	3.034870	0.023796	6.119882
1	5.890652	2.052890	2.771364
1	6.212983	0.399405	0.953866
1	4.588558	-1.448332	0.666682
1	-3.800217	-1.909480	6.555821
1	-5.255433	-0.407382	5.223435
1	-4.232451	1.279370	3.720275
1	-2.849403	2.753900	-2.552557
1	-0.224267	5.739887	-0.953394
1	2.227416	1.219832	0.791015
1	3.898161	3.025150	0.578373
1	2.400968	3.967247	-3.334835
1	0.727343	2.150611	-3.124968
1	3.972797	4.408596	-1.477228
1	-1.714843	4.951027	-2.780517
1	0.157479	4.333849	1.039078
1	-2.470268	1.356561	-0.549880
1	-1.782175	1.485746	3.558754
1	-1.339655	-1.735544	6.363063
1	3.922315	1.876768	4.269251
1	2.641701	-1.636669	2.190971

MC-c

8	0.000000	0.000000	0.000000
7	0.000000	0.000000	4.195349
7	1.689856	0.000000	2.792513
7	-0.326293	1.202724	2.180477
7	-0.274831	2.526514	-0.006991
6	0.390841	0.486495	2.950401
6	1.020557	-0.770452	4.750924
6	2.050820	-0.773280	3.897544
6	2.555183	0.278288	1.706360
6	2.762110	1.589004	1.283635
6	3.608982	1.835592	0.209622
6	4.263319	0.788207	-0.429126
6	4.063010	-0.517745	0.006024
6	3.204219	-0.775992	1.066254
6	-1.292660	0.138015	4.759011
6	-1.860626	-0.943760	5.429437
6	-3.109350	-0.813244	6.022675
6	-3.804427	0.386193	5.934163

6	-3.238829	1.456992	5.251099
6	-1.984608	1.344020	4.667539
6	-0.110854	2.590948	-1.358742
6	-0.959643	1.921115	-2.269699
6	-0.727130	1.956591	-3.639601
6	0.351588	2.659273	-4.161573
6	1.185432	3.353280	-3.283742
6	0.959221	3.326842	-1.918028
6	0.763499	-0.084909	-0.971785
5	-0.203824	1.468548	0.804086
1	0.931499	-1.203402	5.731497
1	3.033313	-1.202961	3.986022
1	3.758992	2.855044	-0.124126
1	4.925004	0.989127	-1.263397
1	4.563077	-1.341285	-0.490067
1	-3.543421	-1.659025	6.543418
1	-4.782714	0.484753	6.389893
1	-3.775099	2.395687	5.173108
1	-1.398341	1.422493	-4.304848
1	2.022794	3.924239	-3.673385
6	0.720608	-1.189821	-1.907386
6	1.607150	-1.175678	-2.987191
6	-0.191419	-2.238759	-1.750219
6	1.583086	-2.210181	-3.909912
1	2.298629	-0.347613	-3.100728
6	-0.208457	-3.269567	-2.672453
1	-0.873134	-2.225928	-0.908397
6	0.677588	-3.253832	-3.749412
1	2.262607	-2.203631	-4.753278
1	-0.910923	-4.086644	-2.561129
1	0.658197	-4.062876	-4.470751
1	1.523725	0.684753	-1.130826
1	1.607968	3.868563	-1.238282
1	0.531598	2.683730	-5.230127
1	-1.804329	1.367309	-1.873221
1	-1.334975	-1.890575	5.473177
1	-1.546409	2.171104	4.127812
1	2.253950	2.404504	1.781203
1	3.018307	-1.793502	1.390541

MC-t

8	0.000000	0.000000	0.000000
7	0.000000	0.000000	4.276486
7	1.695360	0.000000	2.878982
7	-0.363595	1.113429	2.225869
7	-0.470989	2.408666	0.026788
6	0.381200	0.448755	3.013619
6	1.043391	-0.706874	4.870253
6	2.077397	-0.706771	4.022055
6	2.576696	0.270745	1.803153
6	2.674968	1.550566	1.263616
6	3.582483	1.792611	0.240759
6	4.407799	0.778506	-0.231050
6	4.310921	-0.495380	0.317306
6	3.388303	-0.754752	1.322036
6	-1.298547	0.119943	4.829928
6	-1.841642	-0.958482	5.525734
6	-3.096086	-0.845395	6.110685
6	-3.821140	0.333178	5.988870
6	-3.279813	1.400840	5.281366
6	-2.020579	1.305291	4.705539
6	-0.503772	2.389866	-1.328512
6	-1.670185	2.035342	-2.043235
6	-1.672202	1.931676	-3.432402
6	-0.510968	2.149035	-4.155312
6	0.650873	2.526704	-3.472128
6	0.652401	2.664884	-2.099491
6	-0.542795	-0.380942	-1.057800
6	0.234450	-0.853186	-2.184352
6	1.633072	-0.838011	-2.128905
6	2.366389	-1.273814	-3.215626
6	1.711812	-1.720465	-4.364128
6	0.323392	-1.725474	-4.428525
6	-0.417988	-1.287249	-3.340491
5	-0.331435	1.354084	0.843190
1	-1.632987	-0.418571	-1.122766
1	0.964148	-1.100550	5.868131
1	3.075338	-1.091332	4.137709
1	3.653727	2.789899	-0.177284
1	5.120544	0.979721	-1.022137
1	4.942863	-1.297003	-0.047346
1	-3.510501	-1.688373	6.651610
1	-4.803481	0.418311	6.438612
1	-3.839121	2.323661	5.178919

1	-2.588736	1.658097	-3.945570
1	1.565673	2.705746	-4.027940
1	2.120940	-0.474373	-1.233212
1	3.449389	-1.262511	-3.176122
1	2.290430	-2.059587	-5.215984
1	-0.178865	-2.062630	-5.327159
1	-1.501569	-1.271798	-3.384302
1	-0.503619	2.046375	-5.234159
1	1.556564	2.943973	-1.570782
1	-1.600497	2.129941	4.147947
1	-1.290638	-1.889215	5.596081
1	2.045468	2.348129	1.635683
1	3.289704	-1.753767	1.730722
1	-2.581647	1.870206	-1.478076

TS2-c

Imaginary frequency -355.9193 cm-1

8	0.000000	0.000000	0.000000
7	0.000000	0.000000	4.173026
7	1.651619	0.000000	2.726331
7	-0.402458	1.142514	2.132313
7	-0.415650	2.411166	-0.065188
6	0.345611	0.457377	2.904549
6	1.055017	-0.726615	4.724415
6	2.062263	-0.728129	3.843335
6	2.476047	0.264653	1.603547
6	2.655613	1.570128	1.153993
6	3.454489	1.807331	0.042066
6	4.091111	0.754726	-0.606075
6	3.918147	-0.545775	-0.143575
6	3.104644	-0.794194	0.953871
6	-1.277963	0.128762	4.770593
6	-1.803595	-0.943829	5.488585
6	-3.035997	-0.820336	6.116590
6	-3.757237	0.362589	6.016351
6	-3.234293	1.423810	5.285830
6	-1.996759	1.318033	4.666463
6	-0.219163	2.371500	-1.380046
6	-0.821816	1.380294	-2.226520
6	-0.541043	1.337040	-3.612283
6	0.393231	2.170745	-4.164501

6	1.017894	3.141919	-3.342172
6	0.725969	3.245409	-2.010351
6	0.522376	-0.058398	-1.170786
5	-0.284607	1.305300	0.734875
1	1.002454	-1.132554	5.719073
1	3.056884	-1.132221	3.915946
1	3.580403	2.822518	-0.314423
1	4.715977	0.946819	-1.470411
1	4.403137	-1.373278	-0.648074
1	-3.436803	-1.659006	6.674226
1	-4.722747	0.455635	6.499694
1	-3.791375	2.349541	5.198562
1	-1.059709	0.610529	-4.228780
1	1.742184	3.819944	-3.782916
6	0.333027	-1.272034	-1.967953
6	1.167979	-1.497568	-3.063530
6	-0.652849	-2.202955	-1.637073
6	1.011682	-2.642876	-3.829953
1	1.930239	-0.767245	-3.313229
6	-0.807022	-3.345038	-2.407330
1	-1.285928	-2.019792	-0.777266
6	0.022725	-3.565593	-3.503745
1	1.659536	-2.818002	-4.680787
1	-1.573507	-4.067934	-2.153618
1	-0.099950	-4.460708	-4.102780
1	1.415694	0.532085	-1.376825
1	1.199804	3.994412	-1.386511
1	0.631416	2.118990	-5.220161
1	-1.672530	0.828392	-1.844475
1	-1.257553	-1.878506	5.542486
1	-1.592031	2.136600	4.089019
1	2.158335	2.388074	1.658864
1	2.934499	-1.807848	1.297580

TS2-t

Imaginary frequency -148.8803 cm-1

8	0.000000	0.000000	0.000000
7	0.000000	0.000000	4.256369
7	1.801159	0.000000	2.997408
7	-0.183840	1.124448	2.182854
7	-0.013121	2.418328	0.001694

6	0.481204	0.448792	3.028382
6	0.995231	-0.704969	4.931838
6	2.093353	-0.703559	4.168317
6	2.769167	0.307310	2.008953
6	2.886943	1.599908	1.503111
6	3.882350	1.883790	0.577570
6	4.776622	0.898892	0.174154
6	4.655235	-0.389153	0.682943
6	3.645738	-0.690740	1.587396
6	-1.338475	0.118780	4.703048
6	-1.930763	-0.952429	5.369562
6	-3.228400	-0.837666	5.850478
6	-3.947039	0.334749	5.653624
6	-3.355791	1.394735	4.975271
6	-2.054221	1.297755	4.503157
6	-0.170741	2.411583	-1.318316
6	-1.295608	1.789853	-1.936902
6	-1.424972	1.734264	-3.338207
6	-0.413764	2.189638	-4.143989
6	0.720326	2.799027	-3.553583
6	0.831518	2.930065	-2.197484
6	-0.291519	-0.262009	-1.216530
6	0.756153	-0.532813	-2.188382
6	2.098084	-0.338517	-1.845135
6	3.091855	-0.597591	-2.770340
6	2.757900	-1.047635	-4.047235
6	1.426319	-1.237822	-4.396013
6	0.424824	-0.977991	-3.470259
5	-0.086719	1.308921	0.793078
1	-1.302329	-0.600545	-1.438139
1	0.837618	-1.099738	5.919874
1	3.079090	-1.088238	4.362250
1	3.962560	2.888787	0.180649
1	5.558909	1.132473	-0.538682
1	5.337056	-1.169083	0.364345
1	-3.680924	-1.675117	6.368988
1	-4.962454	0.420748	6.022437
1	-3.909208	2.312869	4.814723
1	-2.320757	1.302973	-3.773328
1	1.514559	3.168408	-4.194109
1	2.341721	0.015463	-0.852181
1	4.130056	-0.447949	-2.497431
1	3.539380	-1.249625	-4.771129

1	1.167824	-1.584742	-5.389500
1	-0.616549	-1.119299	-3.737635
1	-0.489083	2.119624	-5.222687
1	1.700009	3.396337	-1.746367
1	-1.593801	2.115775	3.968101
1	-1.383989	-1.879428	5.497514
1	2.199963	2.373159	1.818850
1	3.530674	-1.700378	1.964327
1	-2.144541	1.548151	-1.307561

19-c

8	0.304083	-0.586438	0.490012
7	-3.750854	-0.707655	-0.024559
7	-2.527689	0.392152	1.420559
7	-1.629338	0.045765	-0.796229
7	0.636058	0.829309	-1.520000
6	-2.521910	-0.082608	0.116598
6	-4.468295	-0.626783	1.168957
6	-3.719245	0.045320	2.052700
6	-1.481429	1.134386	2.031117
6	-1.020497	2.305554	1.439405
6	0.017611	3.009029	2.036704
6	0.577856	2.558702	3.227335
6	0.103513	1.392294	3.816892
6	-0.919508	0.672440	3.215195
6	-4.177345	-1.433379	-1.166135
6	-4.864169	-2.632270	-0.989257
6	-5.325717	-3.334880	-2.094648
6	-5.089010	-2.855795	-3.376802
6	-4.389657	-1.665976	-3.546177
6	-3.936005	-0.947026	-2.449088
6	1.900014	0.669246	-1.396269
6	2.519507	-0.304214	-0.392671
6	3.978262	-0.087236	-0.122988
6	4.725447	0.724172	-0.874916
6	4.137152	1.475464	-1.978744
6	2.819700	1.442317	-2.232424
6	1.635761	-0.308175	0.869159
5	-0.238919	0.095425	-0.598940
1	-5.466221	-1.018239	1.256474
1	-3.929737	0.353438	3.061934

1	0.384395	3.915714	1.570167
1	1.385274	3.113230	3.691408
1	0.545940	1.025950	4.735755
1	-5.860146	-4.266625	-1.949462
1	-5.442294	-3.408716	-4.239332
1	-4.196757	-1.286933	-4.543166
1	4.423778	-0.659359	0.684077
1	4.791002	2.090223	-2.588530
6	2.076326	-1.311395	1.906198
6	2.853376	-0.908205	2.988938
6	1.727585	-2.654306	1.783355
6	3.290741	-1.833916	3.929540
1	3.111825	0.140659	3.097717
6	2.159675	-3.580386	2.724003
1	1.100559	-2.963914	0.955287
6	2.945063	-3.173518	3.797822
1	3.895037	-1.507643	4.768680
1	1.880120	-4.623030	2.621340
1	3.280706	-3.897227	4.532016
1	1.675545	0.694234	1.317966
1	2.368831	2.031633	-3.022385
1	5.786590	0.832536	-0.681279
1	2.425453	-1.310954	-0.834248
1	-5.022656	-3.022783	0.009241
1	-3.379842	-0.029023	-2.575322
1	-1.460631	2.648884	0.511382
1	-1.264211	-0.262310	3.640813

19-t

8	0.103145	0.436154	0.283740
7	-0.095024	-0.036018	4.301961
7	1.651399	-0.081521	2.987313
7	-0.124180	1.464597	2.454247
7	-0.538498	2.826603	0.400377
6	0.425557	0.535085	3.151672
6	0.795404	-0.981076	4.810302
6	1.866591	-1.005789	4.005784
6	2.615372	0.247838	1.993413
6	3.115655	1.542307	1.918300
6	4.088413	1.841207	0.974358
6	4.561324	0.853528	0.118444

6	4.047738	-0.434629	0.193564
6	3.067498	-0.739302	1.127927
6	-1.387299	0.212347	4.831647
6	-2.126520	-0.850338	5.346040
6	-3.375531	-0.616374	5.906475
6	-3.897738	0.670372	5.939883
6	-3.160379	1.723872	5.410732
6	-1.905018	1.505250	4.860814
6	-0.981126	2.758758	-0.797568
6	-1.254011	1.420105	-1.472656
6	-1.509516	1.481396	-2.946672
6	-1.640860	2.639925	-3.595159
6	-1.511021	3.910493	-2.888829
6	-1.213799	3.970926	-1.580928
6	-0.125416	0.425695	-1.109123
6	1.151322	0.644624	-1.904254
6	1.891645	1.824225	-1.819017
6	3.026194	2.001310	-2.597575
6	3.453650	0.997819	-3.459662
6	2.737150	-0.189045	-3.535990
6	1.593087	-0.358159	-2.764033
5	-0.174507	1.566199	1.056749
1	-0.489532	-0.577164	-1.354343
1	0.595385	-1.514555	5.722458
1	2.784695	-1.562823	4.072834
1	4.478767	2.850260	0.909846
1	5.314710	1.092478	-0.622365
1	4.395212	-1.199338	-0.490805
1	-3.945053	-1.447617	6.305862
1	-4.875797	0.850774	6.370394
1	-3.562023	2.730472	5.428165
1	-1.605326	0.536545	-3.473519
1	-1.642970	4.826372	-3.455432
1	1.591171	2.607244	-1.134409
1	3.586842	2.926413	-2.521384
1	4.342474	1.139285	-4.064497
1	3.064238	-0.982837	-4.198454
1	1.033450	-1.286224	-2.831827
1	-1.842738	2.657382	-4.659908
1	-1.085388	4.913718	-1.061669
1	-1.332802	2.315424	4.432129
1	-1.734027	-1.859214	5.293866
1	2.738753	2.302494	2.591430

1	2.636377	-1.731930	1.180878
1	-2.164430	1.020181	-1.000069

20

8	0.343533	-0.550835	0.506113
7	-3.762129	-0.720879	-0.043952
7	-2.562081	0.269089	1.494402
7	-1.649605	0.115816	-0.743526
7	0.613587	0.947606	-1.353416
6	-2.543939	-0.090267	0.155487
6	-4.483465	-0.758675	1.149125
6	-3.748843	-0.152579	2.090500
6	-1.561229	1.023473	2.164577
6	-1.182546	2.268810	1.674704
6	-0.213499	3.001730	2.347840
6	0.361380	2.502570	3.511562
6	-0.025373	1.257833	3.995247
6	-0.979703	0.511330	3.317926
6	-4.166536	-1.365691	-1.240726
6	-4.793541	-2.606904	-1.164355
6	-5.232357	-3.232479	-2.323984
6	-5.032217	-2.633050	-3.560987
6	-4.392965	-1.400193	-3.630959
6	-3.963600	-0.758818	-2.477557
6	1.986808	0.728192	-1.348365
6	2.553158	0.127260	-0.211367
6	3.912255	-0.149885	-0.192559
6	4.725893	0.183723	-1.271982
6	4.165523	0.798891	-2.383660
6	2.802857	1.064097	-2.428581
6	1.615838	-0.106424	0.960521
5	-0.260082	0.179770	-0.507904
1	-5.472273	-1.179024	1.198539
1	-3.965286	0.061651	3.122461
1	0.087483	3.969007	1.963122
1	1.114611	3.079381	4.035515
1	0.433434	0.852986	4.889381
1	-5.719828	-4.198217	-2.257544
1	-5.367492	-3.125766	-4.466092
1	-4.230317	-0.927357	-4.592687
1	4.344242	-0.625118	0.681233

1	4.788615	1.066333	-3.230104
6	2.141158	-1.081473	1.984111
6	2.861002	-0.621038	3.082858
6	1.935622	-2.449576	1.830135
6	3.378720	-1.514735	4.013729
1	3.012303	0.446368	3.211099
6	2.445896	-3.343970	2.762286
1	1.364000	-2.802989	0.980445
6	3.170943	-2.879765	3.855201
1	3.938382	-1.144458	4.865576
1	2.278010	-4.407841	2.635819
1	3.568979	-3.579303	4.581737
1	1.469233	0.861111	1.462947
1	2.362604	1.526544	-3.306406
1	5.786899	-0.033920	-1.240765
1	-4.922305	-3.088970	-0.202256
1	-3.457132	0.194489	-2.524600
1	-1.638874	2.650725	0.769774
1	-1.257682	-0.477430	3.662324
1	0.234969	1.411091	-2.163315

TS-3

Imaginary frequency -440.3637 cm-1

8	0.000000	0.000000	0.000000
7	0.000000	0.000000	2.439834
7	2.540477	0.000000	1.683458
6	-0.497831	-0.326138	3.707888
6	-1.309739	0.584616	4.390125
6	-1.859473	0.247990	5.619899
1	-2.486686	0.964670	6.138755
6	-1.606036	-0.995733	6.189441
6	3.404581	-0.831099	1.172310
6	4.502585	-2.655016	0.432481
1	4.639054	-3.699114	0.212795
7	3.323986	-2.183150	0.998972
6	2.229964	-3.015354	1.393901
6	2.443988	-3.969529	2.382627
6	1.403010	-4.811128	2.752164
1	1.562710	-5.557056	3.521571
6	0.159461	-4.687187	2.142196
1	-0.654821	-5.338669	2.436697

6	-0.042853	-3.721730	1.163227
1	-1.013300	-3.618132	0.693059
6	0.993328	-2.880426	0.774947
5	1.247111	-0.056113	1.966173
6	-0.253533	-1.578716	4.280514
6	-0.798551	-1.905491	5.515181
1	-0.595447	-2.878776	5.948967
6	5.311776	-1.599227	0.249371
1	6.307664	-1.534412	-0.151514
7	4.637761	-0.473870	0.709177
6	5.143349	0.858507	0.642851
6	5.617569	2.965444	1.677907
1	5.581259	3.612139	2.546424
6	6.182341	3.420535	0.491675
1	6.586196	4.424342	0.433352
6	6.222583	2.588937	-0.620626
1	6.652243	2.941194	-1.550764
6	5.695290	1.306161	-0.551434
6	-0.911975	-0.096518	0.878725
1	-1.358601	-1.090538	1.091002
6	-1.936731	0.997497	1.025198
6	-1.659807	2.269802	0.532757
6	-3.153488	0.754063	1.654091
6	-2.583400	3.293625	0.685890
1	-0.712904	2.427620	0.029125
6	-4.082829	1.777663	1.807234
1	-3.369448	-0.239809	2.034578
6	-3.796567	3.050037	1.326863
1	-2.363448	4.284444	0.303162
1	-5.028599	1.582461	2.300733
1	-4.518989	3.850367	1.444782
1	-2.034981	-1.253735	7.151054
1	0.362468	-2.291398	3.742549
1	-1.500424	1.551756	3.940374
1	5.696418	0.661079	-1.422322
1	4.657915	1.318165	2.680662
1	3.415591	-4.044573	2.857348
1	0.825439	-2.095539	0.042169

21

8	-0.516820	0.821401	2.792660

7	0.651910	0.082356	1.243364
7	-1.589949	1.271890	0.532235
6	1.356525	-0.662940	0.322624
6	1.142245	-0.456868	-1.046766
6	1.811526	-1.228192	-1.983097
1	1.627784	-1.058567	-3.038732
6	2.714502	-2.209152	-1.582242
6	-2.168159	0.696232	-0.456526
6	-3.076318	-0.821946	-1.892297
1	-3.329911	-1.800473	-2.260540
7	-2.369512	-0.653162	-0.703060
6	-1.934189	-1.713461	0.138600
6	-1.156472	-2.733950	-0.396361
6	-0.713530	-3.760878	0.425396
1	-0.095350	-4.547499	0.009895
6	-1.030536	-3.758596	1.778283
1	-0.668760	-4.552166	2.421673
6	-1.811895	-2.736230	2.306013
1	-2.068332	-2.732022	3.358933
6	-2.278629	-1.717322	1.486628
5	-0.612637	0.778635	1.386085
6	2.283417	-1.630165	0.723797
6	2.948704	-2.394963	-0.224694
1	3.659334	-3.145354	0.104678
6	-3.335864	0.395163	-2.387230
1	-3.841797	0.690798	-3.289073
7	-2.780744	1.335334	-1.518353
6	-2.860680	2.741108	-1.695178
6	-1.749746	3.548411	-1.467981
6	-1.848579	4.917581	-1.672025
1	-0.983966	5.545160	-1.489981
6	-3.040020	5.483073	-2.112233
1	-3.108606	6.552578	-2.272697
6	-4.143755	4.670836	-2.340349
1	-5.079710	5.102824	-2.674867
6	-4.060577	3.301620	-2.123957
6	0.678489	0.016232	2.694683
1	0.473383	-0.991209	3.079595
6	1.876975	0.613685	3.380293
6	2.490244	1.754867	2.867036
6	2.373482	0.035338	4.543499
6	3.583261	2.311306	3.514286
1	2.110200	2.194675	1.951790

6	3.469442	0.591705	5.195073
1	1.899766	-0.856110	4.943353
6	4.074567	1.730902	4.680795
1	4.057666	3.197838	3.108848
1	3.850091	0.134600	6.101419
1	4.930675	2.165635	5.184383
1	3.237005	-2.809360	-2.317702
1	2.474277	-1.787179	1.779425
1	0.442140	0.307519	-1.364537
1	-4.928764	2.670026	-2.273116
1	-0.826596	3.104361	-1.122561
1	-0.870601	-2.700604	-1.440509
1	-2.888500	-0.918885	1.890323

4

6	-2.783357	0.279770	0.000000
8	-1.627230	0.279770	0.000000
8	-3.939484	0.279770	0.000000

TS-4

Imaginary frequency -288.1675 cm-1

8	0.000000	0.000000	0.000000
7	0.000000	0.000000	2.460585
7	2.618967	0.000000	2.048076
6	-0.651878	-0.038152	3.703556
6	-0.212197	0.753424	4.769877
6	-0.843755	0.691604	6.004929
1	-0.483712	1.309431	6.820467
6	-1.937591	-0.145131	6.193217
6	3.453806	-0.962818	1.741161
6	4.372476	-2.957166	1.244717
1	4.389781	-4.021558	1.090676
7	3.205093	-2.293624	1.595571
6	1.912855	-2.876345	1.794024
6	1.529547	-3.243999	3.078430
6	0.251997	-3.743397	3.283942
1	-0.065265	-4.015523	4.283001
6	-0.623877	-3.871789	2.211606

1	-1.626090	-4.249177	2.377279
6	-0.228295	-3.502080	0.932373
1	-0.919570	-3.583811	0.102748
6	1.047764	-2.998605	0.716184
5	1.308956	-0.033225	2.253272
6	-1.755046	-0.873775	3.896984
6	-2.390102	-0.919879	5.130380
1	-3.246562	-1.572451	5.262279
6	5.348059	-2.034732	1.176404
1	6.396792	-2.131236	0.958360
7	4.781584	-0.802344	1.488064
6	5.482461	0.440624	1.480383
6	5.361561	1.317638	2.551333
6	6.067947	2.512106	2.529182
1	5.973021	3.202381	3.358826
6	6.896100	2.820395	1.455802
1	7.446988	3.753363	1.446477
6	7.012262	1.933581	0.392544
1	7.648921	2.172518	-0.450832
6	6.298897	0.742261	0.397136
6	-0.875655	-0.045052	0.814620
1	-2.436190	-0.187783	7.154757
1	-2.104850	-1.475960	3.070384
1	0.627075	1.424519	4.617486
1	6.362570	0.057573	-0.440484
1	4.714854	1.069396	3.382171
1	2.221286	-3.118671	3.903101
1	1.358931	-2.668260	-0.266578
8	-2.043534	-0.109612	1.017496

22

8	-0.374620	1.099320	2.767365
7	0.633931	0.169599	1.223884
7	-1.700079	1.255571	0.592546
6	1.338485	-0.608380	0.305828
6	1.112040	-0.403497	-1.056247
6	1.748474	-1.202911	-1.994242
1	1.560767	-1.039105	-3.049633
6	2.625908	-2.202064	-1.587065
6	-2.204566	0.672412	-0.446416
6	-3.027358	-0.852948	-1.907874

1	-3.260366	-1.835053	-2.279740
7	-2.378763	-0.672282	-0.689044
6	-1.932588	-1.725488	0.160916
6	-1.140050	-2.735442	-0.370908
6	-0.681167	-3.750520	0.456612
1	-0.051487	-4.529960	0.045122
6	-0.995820	-3.744011	1.809860
1	-0.617557	-4.524931	2.458746
6	-1.793349	-2.732126	2.332614
1	-2.044218	-2.724776	3.386441
6	-2.277959	-1.725754	1.508310
5	-0.641934	0.885773	1.367459
6	2.231574	-1.596524	0.719237
6	2.867861	-2.384715	-0.229759
1	3.557559	-3.154302	0.098559
6	-3.268864	0.361550	-2.421898
1	-3.737522	0.654670	-3.344500
7	-2.761291	1.304367	-1.529672
6	-2.835258	2.714029	-1.707671
6	-1.710153	3.506708	-1.509673
6	-1.801219	4.877518	-1.706474
1	-0.926860	5.497283	-1.547075
6	-3.000364	5.452833	-2.111476
1	-3.063925	6.523329	-2.266737
6	-4.117991	4.652264	-2.312707
1	-5.057924	5.094595	-2.620896
6	-4.041688	3.281595	-2.102955
6	0.770064	0.350816	2.594692
1	3.125075	-2.824341	-2.320520
1	2.411079	-1.744027	1.776359
1	0.431453	0.379403	-1.372505
1	-4.916968	2.655344	-2.231487
1	-0.778784	3.053635	-1.197974
1	-0.853893	-2.704081	-1.415099
1	-2.903426	-0.936824	1.907016
8	1.559758	-0.001230	3.413678
